# Recent Advances in Dielectrophoretic Manipulation and Separation of Microparticles and Biological Cells

**DOI:** 10.3390/bios14090417

**Published:** 2024-08-27

**Authors:** Junzhu Yao, Kai Zhao, Jia Lou, Kaihuan Zhang

**Affiliations:** 1 Liaoning Key Laboratory of Marine Sensing and Intelligent Detection, Department of Information Science and Technology, Dalian Maritime University, Dalian 116026, China; 2 2020 X-Lab, Shanghai Institute of Microsystem and Information Technology, Chinese Academy of Sciences, Shanghai 200050, China

**Keywords:** dielectrophoresis, microfluidics, cell separation, integrated, microparticle manipulation

## Abstract

Dielectrophoresis (DEP) is an advanced microfluidic manipulation technique that is based on the interaction of polarized particles with the spatial gradient of a non-uniform electric field to achieve non-contact and highly selective manipulation of particles. In recent years, DEP has made remarkable progress in the field of microfluidics, and it has gradually transitioned from laboratory-scale research to high-throughput manipulation in practical applications. This paper reviews the recent advances in dielectric manipulation and separation of microparticles and biological cells and discusses in detail the design of chip structures for the two main methods, direct current dielectrophoresis (DC-DEP) and alternating current dielectrophoresis (AC-DEP). The working principles, technical implementation details, and other improved designs of electrode-based and insulator-based chips are summarized. Functional customization of DEP systems with specific capabilities, including separation, capture, purification, aggregation, and assembly of particles and cells, is then performed. The aim of this paper is to provide new ideas for the design of novel DEP micro/nano platforms with the desired high throughput for further development in practical applications.

## 1. Introduction

Microfluidics is a scientific and technological field dedicated to the precise control of small volumes of fluids. Using microfabricated channels or chamber structures on microfluidic chips, researchers can achieve fine control over liquids. This technology was first applied in the field of microgas chromatography by Terry et al. in 1975 [1]. This system demonstrated high resolution and sensitivity, enabling the simultaneous separation and detection of small sample quantities. The introduction of micrototal analysis systems in 1990 garnered widespread attention and spurred the rapid development of microfluidics [2]. Microfluidics utilizes microscale fluid channels and tiny droplets to control and manipulate fluids. It is a method of handling and operating fluids at the micrometer scale, effectively shrinking liquid handling processes from traditional laboratory settings [3]. By directing liquids into small channels and chambers, microfluidic technology takes advantage of microscale fluid properties, such as surface tension [4], microfluidic effects, and micromixing [5], to achieve precise control over fluids. One significant advantage of microfluidics is its ability to control fluids at very small scales, making experimental processes more efficient and flexible. It allows for high-throughput processing of samples, reduces the consumption of reagents and samples, and enables the integration of highly automated experimental systems [6]. Over the past two decades, microfluidic devices, particularly those based on polydimethylsiloxane (PDMS) soft lithography, have been widely used for particle focusing [7], capturing [8], concentration [9], and separation [10], ranging from nanoscale to microscale, biological to synthetic, and rigid to flexible. Microfluidic technology finds broad applications in various fields, including biology, chemistry, medicine, and environmental science [11,12].

In the field of microfluidics, various methods are available for the manipulation and separation of particles or cells. These methods use different physical principles [13], such as acoustic [14], electric [15], and magnetic fields [16], to achieve precise control and separation of particles or cells, as shown in Table 1. Acoustic separation is achieved by generating pressure waves in tiny channels. These oscillating waves trigger the lateral movement of the particles and achieve separation. This utilizes the differences in density and compressibility between the particles in the microchannel and the surrounding fluid. Rapid, minimally invasive, and selective separation of microparticles can be achieved [17]. Electrophoretic separation uses an electric field to apply force to particles or cells with different electrical characteristics, causing them to polarize and migrate under the force of the electric field. This allows various particles or cells to be guided to specific locations based on their electrical characteristics, resulting in efficient and precise separation [18]. Magnetic separation manipulates and separates magnetic particles or cells by applying a gradient magnetic field. When these particles or cells are exposed to a gradient magnetic field, a magnetic force is generated that drives them in the direction of the gradient. Varying the magnetic field gradient effectively isolates particles or cells with different magnetic properties within a microfluidic channel [19]. Optical separation manipulates particles or cells by means of radiation pressure exerted by a focused beam. When these particles or cells are subjected to a focused beam within a microchannel, a force is generated that moves them from the fluid to a precise location. Selective separation can be achieved by adjusting the intensity, direction, and focus of the beam according to the optical properties of the particles or cells. Using the radiation pressure induced by the focused light, particles or cells can be effectively manipulated and localized in a microfluidic environment [20].

In 1951, Herbert Pohl first observed and documented the phenomenon of dielectrophoresis (DEP), which describes the motion of suspended particles in the presence of a non-uniform electric field. In his pioneering experiments, Pohl applied voltages of up to 10 kilovolts to generate an electric field strong enough to achieve the experimental goal [35]. Although such voltage levels hindered the practical development of DEP devices, the situation significantly improved in the 1990s with the advancement of microfabrication techniques. These microsystems operated at much lower voltages, leading to the development of useful and practical DEP microdevices [36]. DEP is a highly effective technique extensively applied in microfluidic separations, harnessing the electrophoretic migration of electrically neutral microparticles or cells within a non-uniform electric field [37]. The strength and direction of the DEP force depend on the particle size and dielectric characteristics. This enables DEP technology to effectively separate particles of comparable sizes with varying dielectric properties. DEP technology involves the application of a non-uniform electric field to generate an electrophoretic force that drives the movement of particles or cells. The direction and strength of this force depend on the dielectric properties of the particles and variations in the electric field gradient. There are differences in the dielectric constants of different materials, and the DEP technique is effective in separating particles or cells composed of different materials [38]. As a non-contact separation method, DEP avoids damage or deformation that may result from physical contact [39]. In addition, the DEP technology is highly sensitive and capable of manipulating and separating tiny particles or cells [40]. By adjusting the frequency and intensity of the electric field, precise, selective manipulation of particles or cells can be achieved. It is cost-effective and suitable for various laboratory and practical application scenarios. Continuous research by researchers on the principles of dielectric electrophoresis and microfluidics has enhanced the performance of dielectric electrophoresis technology, making it widely used in a variety of fields such as cell processing, separation, and particle sorting. Nowadays, DEP is capable of operating and separating with nanometer and micrometer precision.

## 2. Dielectrophoresis

Dielectrophoresis (DEP) is the movement of dielectric particles in a non-uniform electric field due to the interaction of the dipoles induced by the particles in the non-uniform electric field with the spatial gradient of the electric field. When a particle is placed in a non-uniform electric field, different forces are generated on each side of the particle. The difference in magnitude of the forces on each side of the particle then produces a net force known as the DEP force. The direction of the DEP force depends on the polarizability of the particle and the suspension medium. For a uniform spherical particle, the generalized expression for the DEP force is given by [41,42]

(1) FDEP=2πεmr3Re⁡fCM∇E|2

where $\varepsilon_{m}$ describes the dielectric permittivity of the suspending solution, r is the radius of the particle, $\nabla |E{|}^{2}$ is the gradient of the square of the electric field, Re indicates the real part of the Clausius-Mossotti (CM) factor ($f_{CM}$) which is a function of the frequency of the electric field and dependent on the dielectric properties of the particle and the suspending medium. The CM factor is expressed as:

(2) $$f_{CM}=\frac{\varepsilon_{p}^{*}-\varepsilon_{m}^{*}}{\varepsilon_{p}^{*}+2\varepsilon_{m}^{*}}$$

where $\varepsilon^{*}=\varepsilon -(j\sigma /\omega )$, $\varepsilon_{p}^{*}$ and $\varepsilon_{m}^{*}$ describe the complex permittivity of the particle and the suspending medium, respectively. σ signifies the electric field conductivity, ω denotes the angular frequency, and $j=\sqrt{-1}$. If the particle’s polarization exceeds that of the medium, indicated by $\left(\varepsilon_{p}^{*}-\varepsilon_{m}^{*}\right)>0$, $f_{CM}>0$, the particle experiences a positive dielectrophoretic (pDEP) force, drawing it toward regions of elevated electric field intensity. Otherwise, when the particle’s polarization is less than the medium, as shown by $\left(\varepsilon_{p}^{*}-\varepsilon_{m}^{*}\right)<0$, $f_{CM}<0$, the particle is subjected to a negative dielectrophoretic (nDEP) force, propelling it away from zones of high electric field intensity. Consequently, the CM factor is pivotal in ascertaining the orientation of the DEP force exerted on particles. However, since biological cells contain structures such as the cell wall, cytoplasm, and cell membrane, a double-layered spherical shell model can be employed:

(3) $$f_{CMcell}=\frac{\varepsilon_{cell}^{*}-\varepsilon_{m}^{*}}{\varepsilon_{cell}^{*}+2\varepsilon_{m}^{*}}$$

(4) $$\varepsilon_{cell}^{*}=\varepsilon_{wall}^{*}\frac{{\left(\frac{r_{wall}}{r_{mem}}\right)}^{3}+2(\frac{\varepsilon_{cyt+mem}^{*}-\varepsilon_{wall}^{*}}{\varepsilon_{cyt+mem}^{*}+2\varepsilon_{wall}^{*}})}{{\left(\frac{r_{wall}}{r_{men}}\right)}^{3}-(\frac{\varepsilon_{cyt+mem}^{*}-\varepsilon_{wall}^{*}}{\varepsilon_{cyt+mem}^{*}+2\varepsilon_{wall}^{*}})}$$

(5) $$\varepsilon_{cyt+mem}^{*}=\varepsilon_{mem}^{*}\frac{{\left(\frac{r_{men}}{r_{cyt}}\right)}^{3}+2(\frac{\varepsilon_{cyt}^{*}-\varepsilon_{mem}^{*}}{\varepsilon_{cyt}^{*}+2\varepsilon_{mem}^{*}})}{{\left(\frac{r_{men}}{r_{cyt}}\right)}^{3}-(\frac{\varepsilon_{cyt}^{*}-\varepsilon_{mem}^{*}}{\varepsilon_{cyt}^{*}+2\varepsilon_{mem}^{*}})}$$

where $\varepsilon_{cell}^{*}$ is the complex permittivity of the cell, with $r_{wall}$ representing that of the cell wall. The cytoplasm possesses its own permittivity, $\varepsilon_{cyt}^{*}$, and the cell membrane’s complex permittivity is indicated by $\varepsilon_{mem}^{*}$. The force exerted on the cell through AC-DEP is contingent upon the dielectric attributes of these cellular components—the cell wall, cytoplasm, and cell membrane. The strength and orientation of this force are governed by the intrinsic electrical characteristics of the cell’s layered structures. This dependency endows AC-DEP with the capacity to not only characterize but also distinguish between various microalgae species based on their unique dielectric responses.

It can be seen that the strength of DEP forces correlates with both the particle diameter and the gradient of the electric field non-uniformity. Particle trajectory alterations, influenced by the exerted DEP forces, result in distinct streaming patterns for particles of varying sizes, facilitating size-dependent particle separation. Furthermore, the DEP forces’ orientation, which is contingent upon the electrical attributes of the particles and the suspending medium—namely, their permittivity and conductivity, as well as the electric field’s frequency—affords precise control over particle manipulation and sorting based on their electrical characteristics. Owing to its rapid analysis, absence of the need for labeling, high sensitivity and selectivity in particle analysis, and minimal sample requirement, DEP has emerged as a prevalent technique for particle manipulation within microfluidic systems [43,44]. To induce the DEP effect, a spatially non-uniform electric field is essential, which can be generated using insulator-based DC-DEP or microelectrode-based AC-DEP [45], which is summarized in Table 2.

### 2.1. DC-DEP

DC-DEP is generally recognized as a highly effective technique for isolating particles based on size. The magnitude of the DEP effects exerted on the particles linearly scales with the particle diameter, resulting in different DEP forces experienced by mixed particles of varying sizes and leading to their separation into individual streams. The size of the particles and the intensity of the electric field determine the transverse dielectrophoretic force exerted on them, causing larger particles to be deflected more than smaller ones. As particles traverse the microchannel, they are sorted according to size into distinct transverse positions [85].

In the field of DC-DEP microfluidic technology, researchers are constantly exploring new methods to achieve efficient separation of particles and cells, which brings new breakthroughs in sample processing, clinical diagnostics, etc. Liu et al. report a DC-iDEP (DC insulated base electrophoresis) chip, which consists of a single channel with a series of consecutively smaller, sawtooth-like triangular features. Each group of 27 triangles has the same geometry. The distance between the endpoints of the paired triangles, known as the gap, decreases gradually from 73 microns to 25 microns. Successful high-resolution isolation and characterization of neural stem and progenitor cells (NSPCs) was achieved by successive zigzag triangle structures providing different electric field strengths. By measuring the ratio of electrophoretic mobility (EK) to dielectrophoretic mobility (DEP), different cell types were successfully recognized, demonstrating the effectiveness of this technique in cell separation and subpopulation identification. This study revealed the existence of different subpopulations within NSPCs and observed a consistent trend of NSPC abundance with their known terminal fates in the EK/DEP mobility range. In addition, neurogenic and astrocytic progenitor cell subpopulations were successfully differentiated [46]. 

Shi et al. demonstrated a microchip structure based on the principle of microelectrodynamics, which consists of glass micropipettes with a small conical geometry. The device generates a non-uniform electric field at the tip of the pipette, which creates a dielectrophoretic force and successfully achieves a rapid, label-free separation of nanoparticles and exosomes. The iDEP device can extract extracellular vesicles from 200 μL samples in only 20 min, and the yield can be increased by sample concentration. The iDEP device provides a new liquid biopsy technique for the rapid clinical isolation of small extracellular vesicles [47]. Crowther designed microchannels with a serrated structure, where the channel walls consist of triangular-shaped insulating materials that are equally shaped and interconnected with each other. At the point where the microchannel narrows, i.e., where the two triangles on the channel wall are closest to each other, an opening is formed. Different serotypes of Listeria monocytogenes were successfully distinguished by gates of different sizes, suggesting a promising application of the gradient-iDEP method and physical modeling [48].These findings suggest that DC-DEP has potential clinical applications for particle and cell separation, bringing innovative ideas to the fields of liquid biopsy, diagnostics, and sample processing. Sun et al. demonstrated a piezoelectric-enhanced PDMS microfluidic chip that allows for the simultaneous capture and separation of exosomes of different sizes using a DC-iDEP method. The chip comprises a channel with two electrically isolated rear sections, each tailored to induce distinct spatial distributions of non-uniform electric fields, thereby exerting varying dielectrophoretic forces on exosomes in suspension. Applying a potential difference of 2000 V across the main channel length facilitated the size-based separation of exosomes via DEP [49]. Ayala-Mar et al. designed a chip structure that is a microdevice consisting of a main channel and a side channel. The main channel consists of an inlet, an outlet, and two elliptical electrically insulated column regions, and the two arrays of electrically insulated columns capture smaller and larger extracellular vesicles (EVs) based on different gap sizes. Using the iDEP, the authors successfully achieved simultaneous separation and concentration of EVs within a multi-section microfluidic device and divided them into two subgroups based on their particle size. EVs were captured and separated in both sections in just 20 s. And their chip was under electroosmotic flow. The separated particles could be released and collected through the side channels perpendicular to the main channel [50]. 

The microfluidic chip structure designed by Li et al. capitalizes on conductivity differences, utilizing DC voltage to enhance the electric field gradient at the liquid-liquid interface, thereby enhancing the dielectrophoretic force used for particle separation. The electric field gradient within this system is determined by two primary factors: the ratio of the asymmetric orifice sizes and the conductivity difference in the side channels. Experimental findings indicate that as the conductivity ratio of the two electrolyte solutions decreases, the particle separation distance increases, aligning with numerical predictions. Furthermore, numerical simulations reveal that the separation distance expands with higher electric field strength and narrower orifice width. However, higher applied voltage can induce ionic liquid movement, which impacts the separation efficacy. To mitigate this effect, adjustments to the voltage or orifice size can be made [86]. The chip structure designed by Gao et al. uses three pairs of asymmetric holes to apply DC voltage, creating non-uniform electric field gradients that enhance the DC-DEP effect. The channel is composed of three inlet channels and two outlet channels, with the electric field induced by embedded microelectrodes creating a non-uniform field in the asymmetric pore region. It was determined that the PS adsorption thickness had little impact on the DC-DEP behavior but did affect the trajectory movement. By adjusting the conductivity of the suspension within the chip, successful separation of the Chlorella vulgaris microalgal cell population was achieved. This method shows promise for the precise selection of target cells in complex microalgal populations and the removal of unwanted cells from suspensions [51]. The chip structure designed by Song et al. uses local DC dielectrophoretic forces to separate particles and count them. The particles are introduced into the microchannel, and the applied voltage creates an electric field gradient to separate the particles. Particle counting was achieved by detecting changes in the current near the particles. Successful separation of 6 µm and 4 µm particles was achieved with the DEP force moving the 6 µm particles away from the aperture. 4 µm particles produced a signal of 0.004–0.005 V, and 6 µm particles produced a signal of 0.006–0.008 V, with a noise of 0.0004 V. The particle size difference of 0.93 mV can be used for size counting. A single-sided orifice counts and separates particles simultaneously without risk of orifice clogging [52]. 

**Figure 1:**
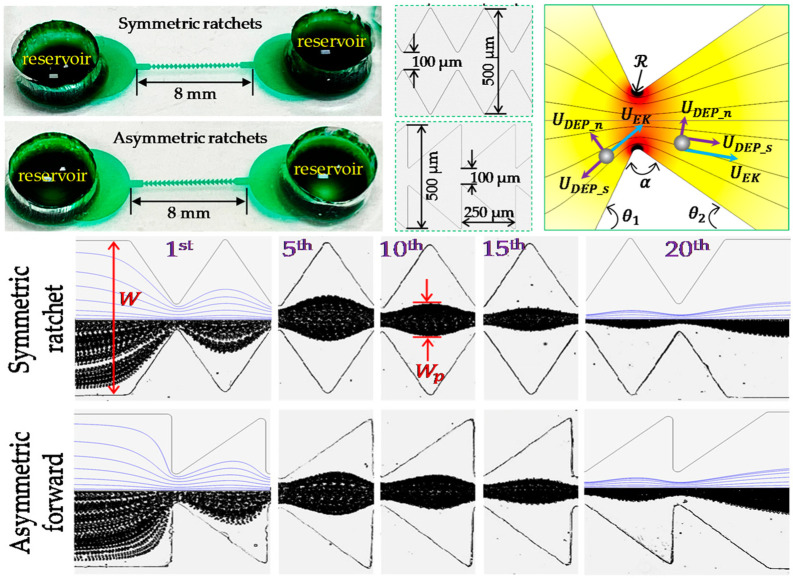
Schematic illustration of the DC-DEP manipulation of particles and cells in a ratchet microchannel. Reproduced from ref. [53]. Copyright 2020, MDPI.

As shown in Figure 1, Lu et al. designed a microchip structure that focuses particles in a ratchet microchannel using DC-DEP. Particle focusing occurs passively via lateral forces induced by the flow and channel structure, directing particles to equilibrium positions in the channel cross-section. Symmetric ratchet microchannels were found to provide better particle focusing than asymmetric ones due to a larger tensor angle. In asymmetric ratchet microchannels, the asymmetry and directional switching of particle DEPs on the upstream and downstream sides of ratchets resulted in stronger forward than backward focusing. However, the ratchet structure has a significant disadvantage: it generates a much higher electric field around the ratchet tip, potentially causing thermal and electrical issues in the sample and microfluidic system [53].

Li et al. designed a chip structure featuring a novel method for particle separation in bifurcated microchannels using a DC electric field. This continuous, sheathless electrodynamic separation utilizes wall-induced electric lift to concentrate particles along the main branch’s centerline, then deflects them into size-dependent flow paths in the side branches. The technique demonstrated label-free separation of 5 µm and 15 µm spherical polystyrene particles along a 2 cm bifurcated microchannel. However, high DC fields can potentially damage biological entities. This risk can be reduced by using DC-biased AC fields [54]. Zhao et al. developed a microfluidic device with nano-orifices that uses DC-DEP to continuously separate similar-sized microns and nanoparticles through pressure-driven flow with varying conductivities. The device successfully separated 140 nm polystyrene from 150 nm magnetic nanoparticles, 470 nm magnetic-coated PS from 490 nm PS nanoparticles, 5.2 μm magnetic-coated PS from 7 μm magnetic-coated nanoparticles, and 14 μm silver-coated hollow glass beads from 15 μm PS particles. Unlike conventional methods, this technique employs asymmetric holes on both sides of the channel wall to create a strong, inhomogeneous electric field, allowing for effective nanoparticle separation while avoiding the Joule heating effect [55].

**Figure 2:**
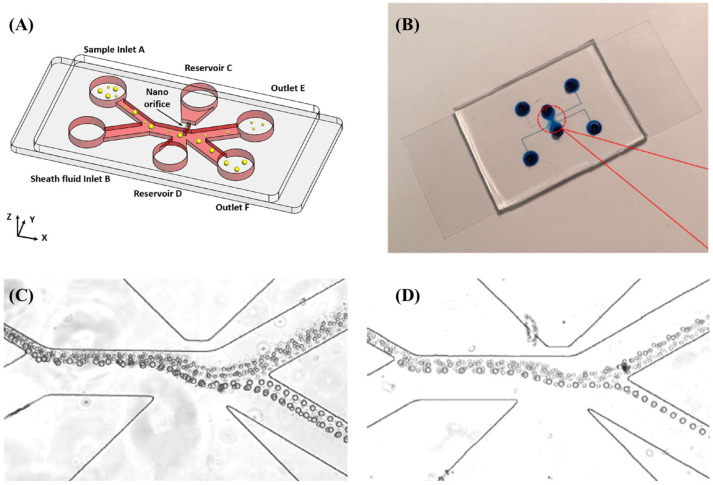
Schematic illustration of (**A**) the nano-orifice-induced DC-DEP manipulation of micro-droplets, (**B**) Example of the nano-orifice based device for the droplets separation, (**C**) Separation of silicone oil droplets with diameter of 9 µm and 14.5 µm under 240 V, (**D**) Separation of silicone oil droplets with diameter of 9 µm and 14.5 µm under 320 V. Reproduced from ref. [87]. Copyright 2018, Elsevier.

Zhao et al. developed a microchip structure utilizing a nanopore-based DC-DEP system to classify oil-in-water emulsion droplets based on their size and content in microchannels. In this DEP system, oil droplets of the same size but varying content are separated through opposite DEP effects: positive DEP and negative DEP. This study successfully achieved size-dependent separation of smaller silicone oil droplets, specifically those measuring 7.5 and 11 µm in diameter, demonstrating high separation resolution with a size difference of only 3.5 µm. The experimental results closely matched the simulation predictions [87], shown in Figure 2. The microchip structure developed by Guan et al. features an innovative serrated microchannel fluidic design that was evaluated for platelet separation and simulated using COMSOL 4.3b. The design utilizes a one-sided zigzag arrangement to ensure a uniform potential distribution within the microfluidic channel. Compared to traditional straight channels, the zigzag microchannel enhances cell separation efficiency. The sharp corners within the zigzag design minimize the horizontal distance required for effective separation and help create an asymmetric DEP electric field. The separation efficiency is highly dependent on the voltage applied; optimal platelet separation was observed at 20 V with an entrance velocity ratio increased from 1:1 to 1:4 [56].

Zhao et al. developed a microfluidic chip with asymmetric orifices to examine the dielectrophoretic behavior of water-in-water ionic liquid (IL) emulsion droplets under a DC electric field. By selecting a suspension medium with the appropriate conductivity, they achieved continuous separation of different types of similarly sized IL droplets. This study discusses and demonstrates the positive and negative DEP behaviors exhibited by IL microemulsion droplets. When the surrounding medium’s conductivity is lower than that of HMIM-PF6 and BMIM-PF6 droplets, both types of IL droplets exhibit positive DEP. Conversely, if the medium’s conductivity is higher than that of the IL droplets, they exhibit negative DEP. Additionally, if the suspension medium’s conductivity is lower than that of BMIM-PF6 droplets but higher than that of HMIM-PF6 droplets, the BMIM-PF6 droplets experience positive DEP, whereas the HMIM-PF6 droplets exhibit negative DEP [88]. Zhao et al. designed a microfluidic device to manipulate and separate polystyrene-based Janus particles and homogeneous polystyrene particles using DC-DEP in microchannels. The device features an asymmetric orifice that creates a strong, non-uniform electric field gradient. By adjusting the suspension solution’s conductivity, one type of particle experiences negative dielectrophoretic (DEP) forces, while the other type experiences positive DEP forces. This mechanism allows for the separation of 5 µm Janus particles from homogeneous polystyrene particles as well as the differential separation of 3 µm and 5 µm Janus particles. To gain deeper insights into the dielectrophoretic behavior of the Janus particles, this study analyzed the effects of DC-DEP forces and electric fields and the impact of the gold coating’s coverage, thickness, and conductivity on these particles. The findings reveal that Janus particles with more than 50% gold coating coverage are subjected to positive DEP forces, which attract them to areas of maximum electric field intensity [89]. 

Ren et al. proposed a nanopore-based active oil/water separation technique that combines hybrid kinetics and differential pressure. Utilizing an inhomogeneous electric field using insulating nanopores under a DC voltage, oil droplets, which have weaker dielectric properties than wastewater, experience negative DEP forces. When the nDEP force predominates over the electro-osmotic hydrodynamic (EHD) force of the oil droplets’ motion, effective kinetic electrofiltration through the nanopores is achieved. To improve the efficiency of oil/water separation, the relative strengths of the nDEP and EHD forces acting on the oil droplets were optimized by increasing the DC voltage, lowering the surface charge density, and refining the nanopore structure. This study concluded that the electric field within the nanopore system is influenced by both the externally applied DC voltage and the electric field generated by the electric double layer (EDL) [57]. Liu et al. discovered that the particle zeta potential significantly impacts wall-induced uplift. They experimentally investigated the electrokinetic transport of four same-sized polystyrene particles through straight rectangular microchannels in a flowing buffer solution. This study revealed that lateral particle migration due to the electro-lift force strongly depends on the particle zeta potential. Particles with higher electrokinetic mobility exhibited enhanced focusing under identical flow conditions. Additionally, it was observed that electrokinetic particle mobility increased logarithmically with decreasing buffer concentration. This increase was attributed to a reduction in the duration of the electro-lift force’s action, leading to decreased electrokinetic particle focusing [90]. 

Crowther et al. explored the development of an innovative insulator geometry to enhance the separation efficiency of iDEP. This research involved shrinking microchannels with six distinct insulators, where the gate spacing was adjusted after every three gates, creating two sets of gate configurations. The selected gate spacing was designed to mimic the existing insulator design used in current measurements. This configuration aimed to streamline particle movement, minimize the potential for extraneous capture regions, and laterally homogenize the force, all while maintaining a high gradient to facilitate effective separation [91]. Mohammadi et al. introduced an innovative microfluidic device designed for the separation of plasma directly from fresh blood inside a microfluidic conduit, enabling real-time optical inspection of plasma composition without the necessity for preliminary or subsequent processing actions. The microchannel features a series of end branches on either side that initially fill with a droplet of fresh blood measuring 2 μL using capillary forces. These end branches create stagnation zones where an applied electric field traps red blood cells via DEP, preventing further red blood cells from entering the channel. To examine the formation of the stagnation zone and ensure the capture of red blood cells during the initial contraction phase, the designed experiment isolates up to 0.1 μL of plasma from 2 μL of fresh human blood droplets. Image analysis measurements indicate that plasma purity reaches 99% after just 7 min [58]. 

Abdallah et al. have developed a microfluidic device focused on optimizing throughput and enhancing sorting accuracy. This study initially considered various standard device geometries and modeled the applied potential to identify the configuration that maximizes sorting efficiency for separating nanoparticles from microparticles. The optimized design demonstrated a theoretical sorting efficiency of 91.6%, while experimental results showed an efficiency of 93.8% when sorting 500 nm particles from 2.5 μm particles using the optimal voltage scheme. This research also explored the potential for sorting smaller particles, ranging from 1 μm to 100 nm, which could be achieved by adjusting the device’s geometry (specifically the contraction width) and fine-tuning the applied potential [59]. Zhao et al. proposed a nanopore-based DC-DEP technique for the continuous separation of polystyrene (PS) micro- and nanoparticles within a pressure-driven flow. By employing a larger pore aspect ratio and shorter, smaller pores, they created a stronger non-uniform electric field gradient at a lower applied voltage, resulting in a more potent DEP force. Size-based separation of 1 μm and 3 μm PS particles was successfully achieved by adjusting the applied potential. For smaller nanoparticles, the suspension solution’s conductivity was modified such that PS nanoparticles of one size experienced positive DEP while those of another size encountered negative DEP. This technique successfully demonstrated the segregation of 51 nm and 140 nm nanoparticles, as well as the separation of 140 nm and 500 nm nanoparticles [60].

### 2.2. AC-DEP

Different AC-DEP microelectrode configurations are utilized for various applications. Particles exhibit unique dielectric signatures, which are leveraged for targeted separation in AC-DEP microfluidic systems. By adjusting the frequency applied, particles with diverse dielectric properties can be effectively segregated. When the frequency is set between the critical frequencies of two mixed particle types, one type experiences positive DEP and moves towards regions with higher electric field intensity, while the other encounters negative DEP and remains distant from areas with stronger electric fields [92]. 

**Figure 3:**
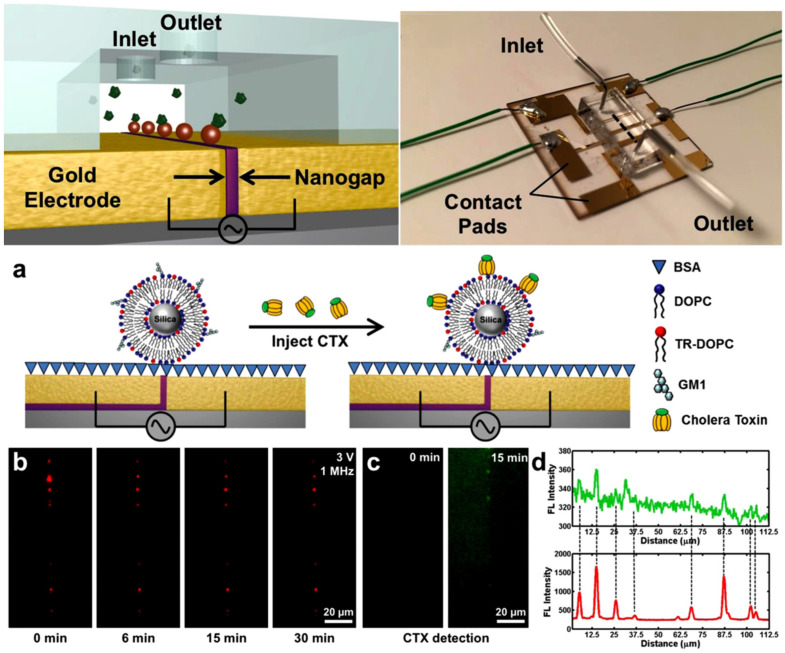
Schematic illustration of the nano-gap electrode-based AC dielectrophoretic microfluidic chip for the trapping of biological particles. (**a**) Application of an AC bias, (**b**) Injection of SSLB particles and trapping by under a bias of 3 V, (**c**) Before and after injection of CTX for 15 min, (**d**) Colocalization of SSLBs with the CTX. Reproduced from ref. [61]. Copyright 2021, Elsevier.

Barik et al. utilized microfluidic channels and nanogap chip architecture in their experimentation. By applying alternating current (AC) bias voltage and modulation of fluid flow rates, they successfully captured and immobilized 700-nanometer-sized SSLB (Solid Supported Lipid Bilayer) particles within nanogaps. They employed DEP to linearly arrange suitable receptors within the nanogaps without necessitating chemical modifications or patterning. Unlike traditional microarrays, the nanogap platform exhibits flexibility across a range of particle sizes, all while ensuring operational voltages are kept at levels that minimize heat and bubble formation [61] (shown in Figure 3). Oshiro et al. introduced an innovative chip design for conducting experiments on cell separation. This chip features a diagonal electrode arrangement that includes both a High-Throughput Dilution (HDF) region downstream and a DEP region. Within the DEP region, applying voltage prompts charged cells to alter their migration direction, swiftly flowing towards the outlet. Unlike similar DEP-based cell separation devices, this chip eliminates the need for buffer exchange before sample loading. Instead, automated buffer exchange within the chip’s HDF region swiftly displaces cells from the sample into the appropriate buffer, ensuring minimal compromise to cell quality. Additionally, the authors successfully achieved target cell separation by manipulating frequency [62]. Hawari et al. designed a new electrode configuration introducing DEP to enhance the harvesting efficiency of marine microalgae. This configuration comprises a central electrode and four symmetrical outer electrodes. The authors employed alternating current modules and AC-DEP modules for microalgae harvesting. Under optimal conditions, the AC-DEP module reduced the aluminum content in the microalgal biomass by 52% compared to the AC module without significant differences in harvesting efficiency. By utilizing the new asymmetric electrode configuration and applying the AC-DEP module for marine microalgae harvesting, a significant reduction in aluminum content was achieved while maintaining high harvesting efficiency [63].

**Figure 4:**
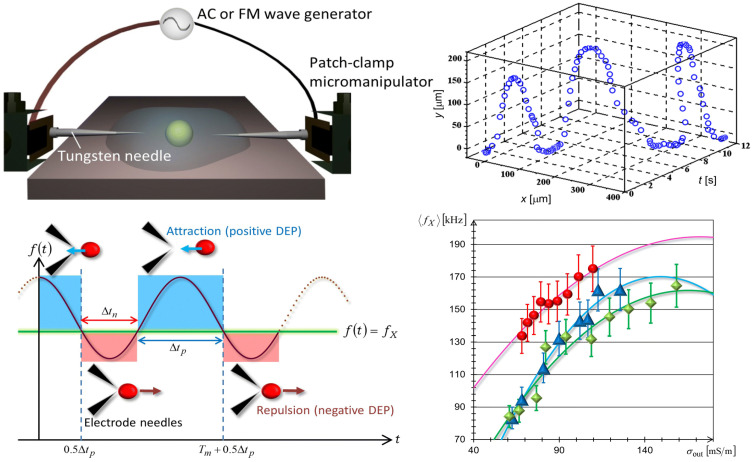
Schematic illustration of the manipulation of vesicles and cells by using frequency-modulated wave dielectrophoresis. Reproduced from ref. [64]. Copyright 2018, Springer.

Frusawa et al. used an inserted microelectrode chip structure in their experiments. The microelectrodes in the chip consisted of tungsten tips that were independently controlled by a controller. Through a plug-in system, an external electric field in the form of AC or FM waves can be applied to the suspension. By adjusting the frequency and amplitude of the electric field, the orientation and manipulation of the cells on the microelectrodes were successfully achieved, and the periodic trajectories of the cells were observed [64], as shown in Figure 4. Malekanfard et al. designed a system employing a low-frequency alternating current electric field to induce an oscillatory electrodynamic flow of either particles or cell suspensions, concentrating them within a ratchet microchannel using induced-charge electrokinetic phenomena (iDEP). The channel consists of two opposing, 20-triangular ratchet teeth arranged along the sidewall. This method involves a trade-off in time to achieve an effectively “infinite” channel length, allowing for the precise concentration of particles and cells under low electric field conditions. Tight focusing of 5 μm particles was achieved after applying a 150 V AC voltage for only 30 s [65]. 

Siebman et al. developed a device that employs AC-DEP to rapidly trap and concentrate green microalga Chlamydomonas reinhardtii on-chip, coupled with fluorescent detection. The device was equipped with four-point needle electrodes arranged orthogonally and spaced 5 mm apart, surrounding a 2 mm-high transparent microfluidic chamber. This configuration was selected for its efficiency in assembling cells into planar 2D structures, facilitating microscope analysis without out-of-focus interference. Upon applying an AC field, algal cells experienced non-uniform electric fields, resulting in positive DEP near the chamber’s bottom, effectively trapping each cell through a combination of the electric field and close packing. The authors successfully utilized AC-DEP to rapidly assemble algal cells into planar, two-dimensional arrays without affecting cell fluorescence [93]. Modarres et al. reported a microfluidic system leveraging frequency-hopping DEP for sorting particles based on size. They exploited the frequency-dependent behavior of particles in response to a non-uniform electric field to manipulate the force field. The device employed interdigitated electrodes to create sinusoidally varying field strengths, enabling particle trapping.

**Figure 5:**
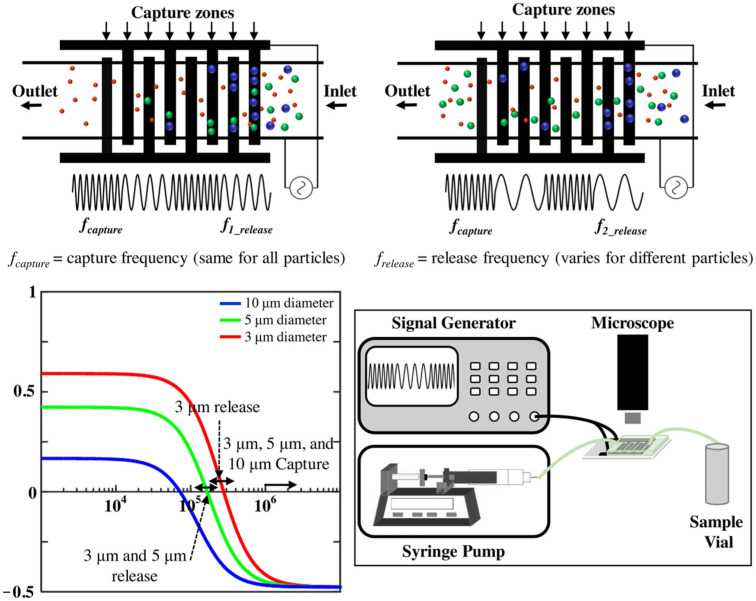
Schematic illustration of the microscale particle and cell enrichment in the microchannel with finger-cross microelectrodes by employing frequency-hoping dielectrophoresis. Reproduced from ref. [66]. Copyright 2019, Elsevier.

By applying specific capture frequencies, particles were directed towards traps located near the electrode digits (nDEP) or edges (pDEP). Adjusting the release frequency allowed for the selective liberation of target particles according to their crossover frequencies. The authors used their device to capture microspheres of 3, 5, and 10 μm with a capture efficiency of 99.8%. They further demonstrated that the device achieved 82.2% enrichment of CTC-like cells [66] (shown in Figure 5). Punjiya et al. designed a microfluidic chip employing a two-electrode geometry consisting of a tangential grounding line and a half-ring trap. This design is capable of applying a uniform electric field in the presence of a steady flow of cells, thereby generating a negative DEP force directed towards the negative Z-axis. This force concentrates the cells at the capture point and is unaffected by gravity. The authors used HEK-293 human embryonic kidney cells to demonstrate a technique that combines negative dielectric trapping with electroporation. The electroporation process was monitored by the efflux of calcium xanthophyll dye while a plasmid encoding red fluorescent protein (RFP) was transfected into the cells. The findings, visualized through fluorescence intensity plots normalized to background levels over time, validated electroporation at a 4 Vp-p amplitude, concurrent with nDEP cell trapping [67]. 

Takahashi et al. designed a continuous cell sorting system leveraging AC-DEP to distinguish cells according to their dielectric characteristics, coupled with a liquid flow control system. Transparent conductive glass was utilized to induce the DEP effect by creating a non-uniform electric field, with an electrode array specifically designed to enhance field non-uniformity. This system was utilized to purify mouse embryonic stem cells and mouse embryonic fibroblasts from a mixed-cell suspension. Initially, the ES-B3 cell purification ratio was approximately 59%, which improved to around 94% after the first cycle of sorting. The purity of ES-B3 cells stabilized at around 90% after 10 sorting cycles. Moreover, the device demonstrated the capability to continuously enhance the purity of mixed-cell suspensions, efficiently processing large volumes in a continuous manner [68]. Derakhshan et al. employed AC-DEP to continuously separate particles and cells within a circular microchannel. Using electrodes positioned along the channel sidewalls, they induced controlled electrothermal flow, reducing fluid mixing compared to conventional interdigitated electrode arrays. Experimental results indicated that a minimum electric potential of 25 V was necessary to separate three polystyrene particles at an average velocity of 200 μm/s. For biological cells, effective separation of white blood cells, red blood cells, and MDA-MB-231 breast cancer cells required a minimum voltage of 9 V. This study found that increasing the applied electric potential beyond 12 V significantly increased electrothermal flow and mixing, while maintaining the potential within 9 V to 12 V achieved effective separation with a temperature rise below 1 K, ensuring suitability for biological applications [69].

**Figure 6:**
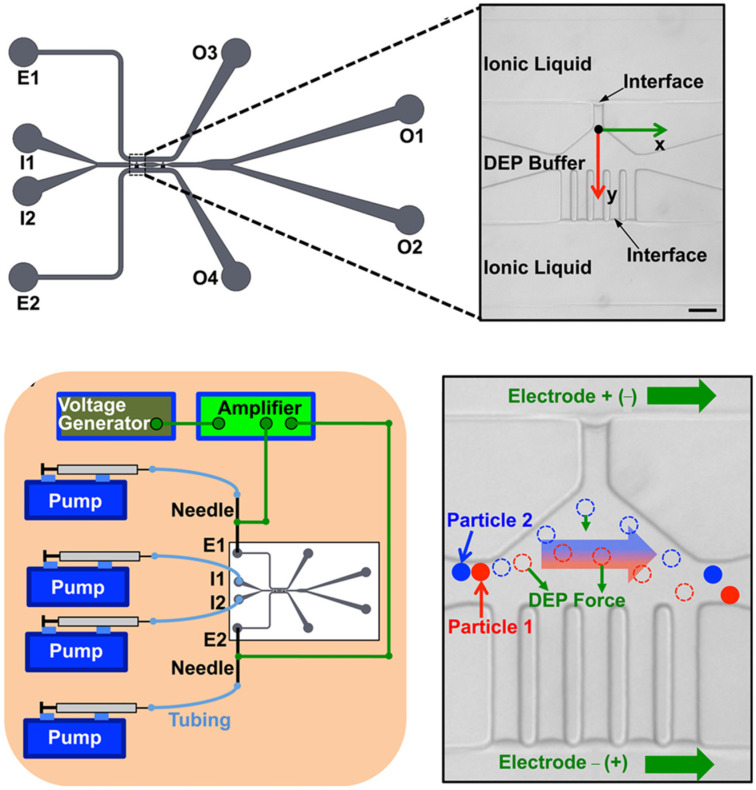
Schematic illustration of the on-chip cell separation based on conductivity-induced dielectrophoresis with 3D self-assembled ionic liquid electrodes. Reproduced from ref. [75]. Copyright 2016, American Chemical Society.

In AC-DEP microfluidic devices, achieving electric field non-uniformity often involves integrating microelectrodes of different geometries and dimensions within the microchannel (shown in Figure 6). Han et al. proposed a method of self-limiting AC-DEP for assembling nanoparticles selectively over large areas. Their circuit design included a lithographically defined capacitor connected in series with a pair of trapping electrodes in each parallel unit. This configuration enabled the capacitor to bear most of the applied voltage when nanoparticles formed bridges across the electrode gap. As a result, the voltage drops across the gap decreased, minimizing the occurrence of multiple nanoparticle trappings at identical sites.

Using this setup, they successfully formed arrays of aligned nanoparticles for further device fabrication and system integration. This self-limiting mechanism resulted in a 787% enhancement in single-particle assembly yield compared to the control, achieving a 70% single gold nanowire (AuNW) assembly yield at 1 MHz and 2 Vp-p [70]. Zhang et al. designed a Y-Y shaped microfluidic device for AC DEP-based isolation of non-small cell lung cancer (NSCLC) cells. The device features a microchannel with alternating triangular protrusions on the upper wall, each of which is electrically charged to create a non-homogeneous electric field. Blood samples containing erythrocytes and circulating tumor cells (CTCs) are introduced from the upper inlet, while a buffer solution is introduced from the lower inlet, directing cells to one side of the channel. At the channel intersection where the streams converge, cells experience dielectrophoretic forces proportional to their sizes due to the varying electric field, facilitating the effective separation of CTCs from erythrocytes. Numerical simulations indicated that with electrical potentials ranging from 1.6 V to 2.2 V and inlet flow rate ratios from 1.9 to 2.5, the separation efficiency could achieve approximately 99% [71]. Lv et al. designed the Taguchi method to design 16 variations of Y-Y microfluidic microchips aimed at optimizing cell capture through AC-DEP for separating circulating tumor cells (CTCs). They performed numerical simulations on flow patterns, electric fields, and cell trajectories, employing the signal-to-noise ratio (SNR) to assess the impact of these factors and determine the most effective configuration. Their findings indicated that increasing the buffer inlet flow velocity enhances separation purity. The sequence of geometric parameters’ impact on separation purity was determined as W > α > L > β, with β having the least influence at 7.81% and W having the most significant impact at 50.48%. The optimal geometric parameter combination was identified as L = 1080 μm, W = 110 μm, β = 60°, and α = 60° [72]. 

Liu et al. proposed an innovative AC-DEP for the direct capture and elimination of cyanobacteria. They explored factors influencing removal efficiency by employing a two-stage process: initially using 30-mesh wire mesh electrodes to capture larger, aggregated cyanobacteria, followed by 80-mesh wire mesh electrodes to capture smaller, filamentous cyanobacteria. In their experiments, a cyanobacteria solution with an initial concentration of 756 μg/L was processed at a flow rate of 0.503 L/h. Applying an AC voltage of 15 V at 10 kHz significantly increased the removal rate from 68.37% without DEP to 89.79% with DEP. Higher initial concentrations of cyanobacteria also enhanced the polarization induction effect, further improving removal efficiency. Optical microscopy confirmed that the captured algal cells maintained their structural integrity, indicating that this DEP method can prevent secondary pollution from reagent addition and phycotoxin release [73]. Jones et al. designed an AC-iDEP sorting device with ionic liquid electrodes for separating different nucleic acid analytes at different outlets within a microchannel. Their device uses DEP forces to guide particles through a constriction zone and sort them by size. The authors performed continuous-flow DEP sorting on four types of double-stranded DNA (dsDNA) analytes ranging in size from 1.0 to 48.5 kilobase pairs (kbp). They found that the instability of the low-frequency electrohydrodynamic directly affected the significant variation of the sorting efficiency, which led to the selective enrichment and exclusion of dsDNA of different sizes at different outlets of the device. Experimental results showed that sorting efficiencies ranged from 0.59 ± 0.04 (effective side exit sorting) to −0.92 ± 0.03 (center exit sorting), achieving sorting efficiencies in excess of 90% [74]. 

Zemánek et al. proposed a novel method for noncontact micromanipulation using controlled AC-DEP. This technique involves modulating the voltage phase shifts applied to electrodes, thereby simplifying hardware requirements and broadening the range of achievable forces. The innovative electrode layout comprises four identical quadrants with parallel, mutually orthogonal electrodes, facilitating arbitrary movement within the manipulation area. Utilizing the negative DEP frequency range, experiments involved 50-micron polystyrene microspheres suspended in deionized water. By alternately applying phase shifts of 0 and π to all electrodes, the control algorithm successfully achieved initial levitation of objects to a height of 140 µm. Both numerical simulations and laboratory experiments confirmed the system’s ability to accurately position and guide a 50 µm microsphere, as well as to bring two objects together and separate them. The worst-case positional error was found to be just 8 µm, representing 16% of the microsphere dimensions or 0.7% of the manipulation span [76]. Li et al. reported a DEP device with an array of bipolar electrodes (BPE) for continuous, specific, and efficient capture of circulating tumor cells (CTCs). Their device transmitted an alternating current field between insulating barriers in parallel microchannels to simultaneously capture CTCs. Embedded along each microchannel wall are microscopic pockets aligned with the tips of BPEs, providing defined volumes for discrete single-cell capture. In their experiments, a mixed cell sample was introduced under a 40 kHz alternating current field at an average linear velocity of 60 μm/s. The researchers observed selective capture and accumulation of breast cancer cells at the BPE tips, while white blood cells passed through without retention [77]. 

Pendharkar et al. designed an insulator-based AC-DEP microfluidic chip for the patterning and fusion of biological cells. The chip has two pieces of ITO glass placed opposite each other with conductive surfaces facing each other within the chip, and the bottom ITO glass is covered with a PDMS film with 6000 microscopic pores for capturing individual cells. By applying an AC electric field, the non-uniform electric field generated by the PDMS layer was utilized to capture and guide the cells to the microwells for pairing. They successfully achieved patterning and cell fusion between CT26 and bone marrow dendritic cells (BMDCs) using iDEP-LC technology. The fusion efficiency was as high as 70% in precisely matched cells, and within four days, 60% of the fused cells were still viable. The authors also investigated the characteristics of the fused cells, including intracellular components labeled with fluorescence and different cell morphologies. This fabrication method provides a straightforward, biocompatible way to generate a large number of PDMS microwells, enabling the chip to efficiently immobilize approximately 6000 cells [78]. Liu et al. reported a generalized membrane antifouling strategy based on AC-DEP in which they prepared Ni/PVDF conductive membranes by electroless nickel plating on PVDF ultrafiltration membranes, which were used as an electrode for constructing a nonuniform electric field at the filtration feed end using an AC electric field. The authors applied their equipment to systematically design flow-through electrofiltration experiments for three colloidal contaminants with different dielectric constants or conductivities. The membrane permeation rate for electronegative SiO_2_ and electropositive Al_2_O_3_ particles, which have relative dielectric constants lower than those of medium water, increased by 90.1% for both membranes when subjected to an AC electric field. This demonstrates the significant enhancement in permeation efficiency under these conditions [79]. 

Zhao et al. described an innovative design for AC-DEP by integrating asymmetrically porous electrodes on opposite sides of a primary microfluidic channel. This setup enables the continuous separation and characterization of particles and droplets. DEP effects manifest within the asymmetric pore region: particles or droplets exhibiting positive DEP move towards smaller pores and deviate from the central axis of the main channel, while those experiencing negative DEP migrate towards the central axis and away from larger pores. The researchers successfully achieved size-based separation of 5 μm and 10 μm polystyrene particles, as well as type-based separation of similarly sized ionic liquid (IL) and oil droplets. They also investigated the migration of particles and IL droplets under various frequencies of the AC electric field. Their study represents the first measurement of the lateral migration of particles and water-in-ionic liquid emulsion droplets in microfluidic chips in relation to the frequency of the applied AC electric field. This study marks the initial exploration of lateral migration of particles and water-in-ionic liquid emulsion droplets in microfluidic devices concerning the frequency of the applied AC electric field, thereby identifying the critical frequency and distinct behaviors of the droplets [94]. 

**Figure 7:**
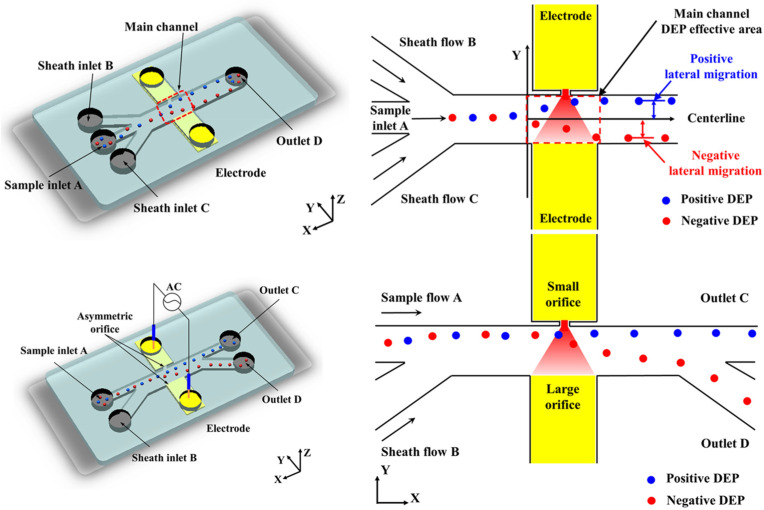
Schematic illustration of the AC-DEP characterization of yeast cells via embedded microelectrodes in the microchannel. Reproduced from ref. [80]. Copyright 2019, American Chemical Society.

Afterwards, they devised a microfluidic DEP chip specifically designed for continuous characterization and separation of viable and non-viable yeast cells, employing a series of asymmetric openings. Positioned horizontally within the main channel are two openings: a narrow 10 μm aperture and a wide 500 μm aperture. Through modulation of AC electric field frequencies, the researchers effectively segregated live and dead yeast cells in deionized water [80], as shown in Figure 7. Wang et al. reported a method for microalgal cell separation utilizing the AC-DEP technique, which incorporates a three-dimensional electrode structure for a DEP separation chip. The electrodes were designed to be bifurcated and located on opposite sides of the microfluidic channel; one side had eight strip electrodes with sharp corners, while the other side contained a rectangular electrode. This arrangement creates a distinct gradient of electric field strength along the electrodes. The authors used this chip to treat a mixed solution containing *Chlorella* and *Clostridium*. They observed that *Closterium* cells were attracted to the strip electrode by positive DEP forces, moved downward through the channel, and were expelled. *Chlorella* cells, on the other hand, were repelled upwards by negative DEP forces, deviated from the strip electrode, and were subsequently expelled. This method was successful in separating *Chlorella* and *Clostridium* cells with an efficiency of more than 90% [81]. 

**Figure 8:**
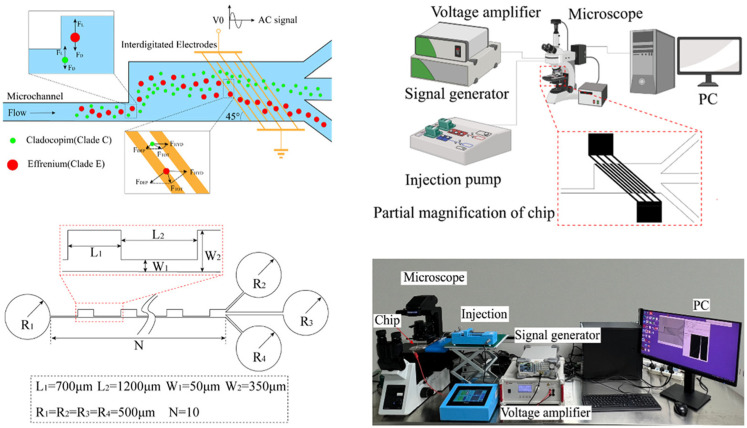
Schematic illustration of the microalgae enrichment by the combination of AC-DEP and inertial microfluidics. Reproduced from ref. [83]. Copyright 2024, AIP Publishing.

Chen et al. used an AC-DEP chip with right-angle bipolar electrodes for the characterization and selection of non-spherical flagellated algae. Compared with the sharp-angled triangular electrode, the electric and flow fields near the right-angled bipolar electrode were milder and more uniform, which favored cell adhesion to the electrode edges at a smaller angle. They simulated the equilibrium state of spherical, elliptical, and spindle-shaped cells under the positive DEP force applied by the right-angle bipolar electrode. The authors applied their chip to successfully achieve 92.06% separation of *Euglena* and *H. pluvialis*. and 92.78% isolation of *Dunaliella salina* and *Platymonas*. A recovery efficiency of 99.06% was achieved in the isolation of Euglena cells with high viability and motility, obtaining 100% purity of live Euglena cells [82]. Zhou et al. developed a microfluidic chip integrating AC DEP and inertial forces for the separation and enrichment of *Symbiodinium*, the microalgae symbiotic with corals. The chip combines a separation module and an enrichment module. In the separation module, *Symbiodinium* cells are first initially stratified by inertial forces, and then DEP forces are used to further separate different cells. In the enrichment module, cell focusing is achieved by controlling the flow rate using inertial contraction-expansion microchannels, and finally, the DEP force directs the focused cells to a single outlet for enrichment. The authors successfully achieved the separation of *Effrenium* and *Cladocopim* cells in *Symbiodinium* using their microchip, with a separation purity of 90%. At the same time, as shown in Figure 8, the concentration of *Symbiodinium* cells at the outlet was approximately 5.5 times the concentration of the original solution, achieving efficient enrichment [83]. 

Julius et al. reported an AC-DEP device using an adaptable dielectrophoresis embedded platform tool (ADEPT). The chip contains six microelectrodes arranged in a circular pattern, each of which can be controlled independently. They first captured cells in the center using a frequency of 200 kHz and an amplitude of 10 Vp-p, and then increased the frequency to 4 MHz and an amplitude of 20 Vp-p to achieve separation of live and dead yeast cells with a separation efficiency of 94%. They further demonstrated that phenotype-based separation experiments were successful in achieving up to 96% separation of yeast and *Bacillus subtilis* [84].

## 3. Applications of DEP

DEP is considered an effective tool for manipulating micro-scaled and nano-scaled targets in the microchannel by using electric fields, which have a wide range of applications, including particle sorting, capture, purification, aggregation, attachment, and localization. These applications have been instrumental in making DEP technology useful in drug screening, cell analysis, biosensors, and microelectronics. Under dielectrophoretic forces, particles or cells move between electrodes in a curvilinear manner due to the electric field strength and spatial distribution, resulting in highly selective sorting. This sorting function can be precisely tuned to the type, size, shape, and charge of the particles, resulting in efficient separation of particle mixtures. Compared to conventional particle manipulation methods, the DEP operation requires a very small sample size, allowing for a non-destructive and highly accurate process.

### 3.1. Particle Separation

A DEP device with self-assembled liquid electrodes consisting of ionic liquids is designed for continuous separation of particles and human cells. This device leverages room-temperature ionic liquid, which exhibits higher conductivity compared with conventional DEP buffers, to form the liquid electrode. By integrating two DEP buffers of varying conductivities with the ionic liquid, the authors managed to harness the applied potential within the primary channel to establish a robust gradient of electric fields. This configuration facilitated efficient separation of polystyrene microbeads and PC-3 human prostate cancer cells, achieving deflection rates of 94.7% for the cells and 1.2% for the microbeads. They also demonstrated the device’s capability to discriminate between viable and non-viable PC-3 cancer cells, achieving deflection rates of 89.8% for viable cells and 13.2% for non-viable cells. Additionally, the device successfully isolated MDA-MB-231 human breast cancer cells from human adipose-derived stem cells (ADSCs), achieving separation purities of 81.8% for ADSCs and 82.5% for MDA-MB-231 cells [75]. 

Wu et al. designed an integrated microfluidic system utilizing bipolar electrodes and surface acoustic wave regions to achieve label-free separation of different types of cells and particles. The system consists of a deterministic lateral displacement (DLD) array for initial separation, a bipolar electrode (BPE) region for particle focusing and separation based on dielectric properties, and a surface acoustic wave (SAW) module that utilizes acoustic radiation force for separation of density and compressibility differences. They injected mixed samples of polystyrene particles, oil droplets, and live/dead yeast cells into a DLD array with an integrated microfluidic chip and performed initial separation based on particle size differences under flow conditions combining a DC electric field and pressure drive. The AC electric field in the BPE region was utilized to generate the DEP force for further focusing and separation of particles based on dielectric properties. In the SAW region, finer separation is achieved by acoustic radiation forces. By adjusting the frequency and voltage of the AC electric field, a separation efficiency close to 90% of the target particles was achieved [95]. Luo et al. introduced a method integrating sheathless prefocusing based on gravitational sedimentation with DEP separation in a microfluidic device for continuous cell sorting according to size or dielectric characteristics. Initially, a tube was inserted into the microfluidic chip’s inlet, allowing gravitational sedimentation with adjustable steering angles within the tube to concentrate cells into a stream at the upstream section of a microchannel. After prefocusing, DEP forces were applied in the microchannel’s downstream area for active cell separation. The researchers successfully demonstrated sorting of yeast cells, approximately 6 µm in diameter, and THP-1 cells, around 13 µm in diameter, based on cell size. Additionally, they achieved separation of OCI AML3 cells and THP-1 cells, both approximately 13 µm in diameter, based on distinct dielectric properties. The technique achieved separation efficiencies exceeding 90% [96]. 

**Figure 9:**
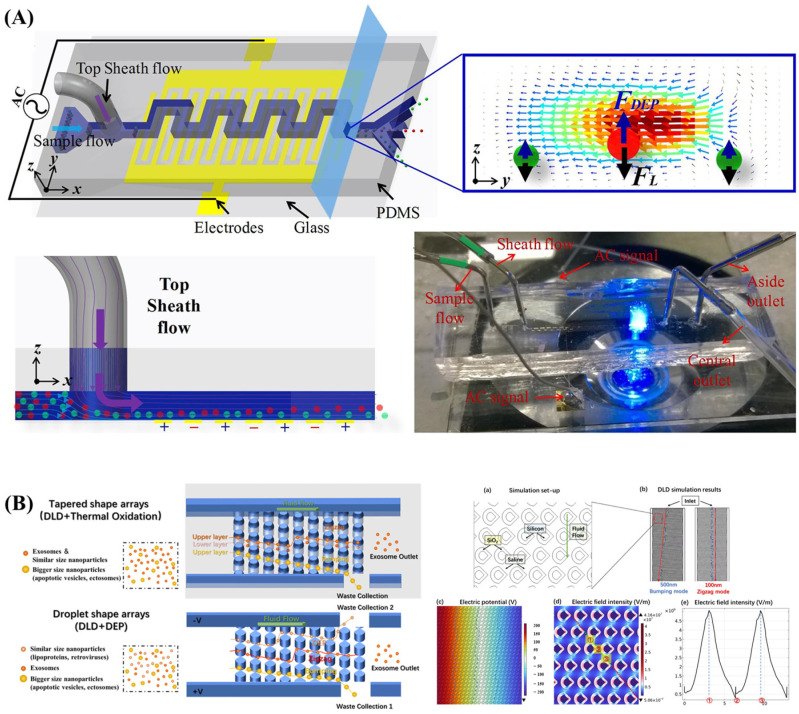
Schematic illustration of tunable separation of particles and exosomes in the hybrid (**A**) dielectrophoresis-inertial microfluidic chip Reproduced from ref. [97]. Copyright 2018, Elsevier; (**B**) dielectrophoresis-deterministic later displacement arrays. Reproduced from ref. [99]. Copyright 2024, MDPI.

As shown in Figure 9A, Zhang et al. presented a hybrid microfluidic platform combining DEP and inertial forces to achieve adjustable particle separation. They employed an external DEP force field alongside inertial forces, using a sheath flow to remove particles from the top inertial focusing positions. This action confined all particles near the microchannel’s bottom, which was patterned with microelectrodes, enabling DEP forces to alter the focusing positions of all particles effectively. The authors successfully demonstrated separation of 13 μm and 5 μm particles with separation efficiencies of approximately 100% and 96%, respectively. Additionally, they conducted experiments to enhance the resolution of particle separation in a binary mixture featuring diameters of 8 μm and 13 μm. By incrementally increasing the applied voltage from 27 volts to 33 volts, the 13 μm particles were focused along the channel centerline, while the 8 μm particles remained concentrated in two streaks near the channel’s sidewalls, effectively achieving separation [97]. Wu et al. introduced a microfluidic platform that integrates driving electrodes for cell and microbead separation alongside bipolar electrodes for individual cell or microbead manipulation using DEP. Their experimental strategy combines pressure-driven flow with deflective DEP barriers to achieve efficient, high-throughput separation. They achieved over 90% recovery efficiencies for yeast cells and polystyrene microbeads. Additionally, they conducted trapping experiments using yeast cells and PS microbeads in solutions of varying conductivity, demonstrating successful capture at the central position of wireless electrodes through negative DEP forces. By applying a rotating electric field, yeast cells underwent translational movement along the periphery of the electrodes, while an array of bipolar electrodes enabled cellular self-rotation influenced by electro-rotational torque and traveling wave DEP forces [98]. 

Wang et al. designed a combination of thermo-oxidative non-uniform deterministic Lateral Displacement (DLD) arrays and an AC-DEP chip for efficient exosome separation. The chip fabricated DLD arrays with nanoscale spacing and tapered structures. They applied AC power, and 600 nm particles were subjected to dielectrophoretic forces and the DLD effect, deviating from the main flow path and being collected from the waste exit. The smaller 100 nm particles flowed directly to the exosome outlet, achieving size-based separation. The authors have successfully used their device to separate exosomes from complex biological samples, achieving a purity of 91.47%, significantly higher than the 57.84% of the original samples [99] (shown in Figure 9B). A microfluidic chip for nanoscale sorting of extracellular vehicles (EVs) was designed by Soong et al. The chip incorporates optically-induced dielectrophoresis (ODEP) technology. They generated a non-uniform electric field by irradiating light through a digital projector on a photosensitive amorphous silicon layer. An AC voltage was used between two ITO substrates to induce a positive ODEP force that attracts the EVs within a defined light pattern. The green light intensity and speed were varied to sort EVs of different sizes from a moving light pattern generated by a digital projector. The authors applied their chip to successfully classify the EVs into three different size categories: small (100–150 nm), medium (150–225 nm), and large (225–350 nm). A separation efficiency of 86% was achieved [100]. Chu et al. also designed microfluidic chips combining the ODEP mechanism with laminar flow patterns in microfluidic systems for sequential size-based particle sorting and separation. They successfully achieved PS separations at 5.8, 10.8, and 15.8 µm using their chip, all with separation efficiencies around 90% [101].

### 3.2. Particle Capture

The atomically sharp edges of monolayer graphene are utilized to generate highly localized electrical field gradients, aiming to trap biomolecules using DEP. Their approach involved creating locally backgated devices that incorporated an 8-nm-thick dielectric layer of HfO_2_ along with graphene deposited via chemical vapor deposition. This configuration produced gradient forces that were ten times stronger than those produced by conventional metal electrodes. This enhancement enabled near-perfect trapping of particles under precise positional control at minimal voltages of only 0.45 V, effectively capturing nanodiamonds, nano-sized beads, and DNA from large-volume solutions within seconds. Their method leveraged the intrinsic thickness of graphene and the precise deposition of insulators using atomic layer deposition (ALD), resulting in a highly scalable and reproducible technique. This approach significantly advanced the precision and reliability of nanoparticle trapping and positioning, thus supporting biological assays at low concentrations and facilitating the investigation of molecular interactions [102]. Ertsgaard et al. developed a novel DEP and surface-enhanced Raman spectroscopy (SERS) platform, termed TRAIL (Trap, Raman, and Imaging Line), for the rapid detection and analysis of nanovesicles. Their experiments focused on utilizing a high-aspect ratio 11-nanometer electrode gap to generate ultra-strong electric field gradients at significantly lower voltages compared to conventional microelectrodes, facilitating efficient DEP line-trapping. The researchers observed that metallic nanoparticles exhibited a positive Clausius-Mossotti factor (CMF) across the standard operating frequency range of 1 Hz to 10 MHz, even in highly conductive buffer solutions. Using 70-nanometer gold nanoparticles, they demonstrated that Raman enhancement could be further optimized by employing gold or silver nanoparticles of varying shapes and sizes. Additionally, functionalizing gold nanoparticles could enable chemically specific trapping in conductive solutions, targeting specific receptor molecules on nanovesicles or analytes, thereby enhancing the trapping specificity and efficiency of the TRAIL platform [103]. 

**Figure 10:**
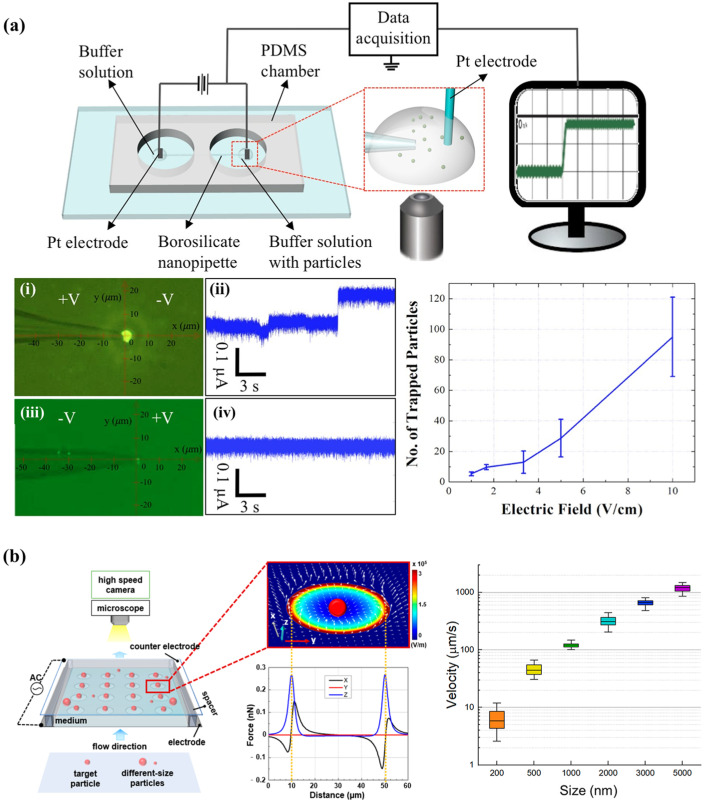
Schematic illustration of selective trapping of particles and exosomes in the (**a**) low-voltage nanopipette dielectrophoretic device, (i) Entrapment under 10 V/cm with the positive bias after 100 s, (ii) Measurement of conductance across the 1 µm pore. (iii) Entrapment of few particles under 10 V/cm applied at the pipette based. (iv) Conductance measurement across the 1 µm pore. Reproduced from ref. [104]. Copyright 2018, Springer Nature; (**b**) dielectrophoretic corral traps. Reproduced from ref. [105]. Copyright 2021, American Chemical Society.

Shi et al. reported a nanopipette-based DEP device designed for the rapid capture of nanoparticles from solutions of various ionic strengths under a low applied DC field. The device effectively trapped particles in the close-proximity region in front of the pipette tip. Optimal trapping conditions were established using a 2-micrometer-diameter pipette and a 10 V/cm positive bias in a 10 mM KCl solution. The capture yield was observed to increase over time, stabilizing after approximately 40 min. The authors successfully captured exosomes from healthy donor plasma and liposomes resuspended in a PBS solution under applied negative potential. Additionally, they demonstrated selective trapping of liposomes, and 510 nm carboxylic acid polystyrene beads were suspended in PBS solution by adjusting the polarity of the voltage on the pipette [104] (shown in Figure 10a). Kwak et al. reported a dielectrophoretic corral trap system for size-selective particle capture, featuring micron-sized circular traps. By applying AC voltage, the DEP electrodes generated non-uniform electric fields. They carefully selected the frequency of the AC voltage, using the CM factor to create conditions where particles with significant susceptibility to the electric field gradient were captured by the DEP gates, while smaller particles passed through unaffected. The system was tested with particles of 2 μm and 3 μm radii to evaluate size-selective trapping efficiency. They found that higher microchannel heights resulted in increased trapping efficiency, contrasting with traditional shallow channel methods. This configuration enabled rapid isolation and concentration of specific-sized particles in a single step, overcoming the inefficiencies of multiple treatments and dilution steps in existing separation techniques [105], as shown in Figure 10b.

A comparative study of a ring electrode system and a point electrode system for capturing particles by DEP was performed by Weber et al. This study focused on assessing response times and the proportion of bacteria responding under identical boundary conditions (applied electric field). Results indicated that the ring electrode system achieved bacterial capture within 1 s, a performance more than 200 times faster than that of the dot electrode system. Additionally, the ring electrode achieved a capture efficiency exceeding 99%. This work underscores the superior efficiency and rapid response of the ring electrode design in DEP-based microbial capture applications [106]. Islam et al. designed a paper-based DEP capture device for capturing micro-scale particles and cells. The device induces a dielectrophoretic force by applying an electric field perpendicular to the direction of fluid flow in this paper channel, using a local non-uniform electric field gradient generated by the insulating fiber structure inside this paper. This force acts on suspended particles and cells, causing them to move out of the fluid and be captured in specific regions of this paper channel. The authors applied voltages as low as 2 V to effectively trap and concentrate micron-sized particles through two mechanisms: constant flow rate or passive capillary flow. They further demonstrated that the device successfully captured fluorescently labeled polystyrene particles and *E. coli* cells [107].

### 3.3. Particle Purification

Weirauch et al. devised an economical mesh-based filter using DEP, employing structured field disruptors for particle purification. The system initially traps a mixed particle sample and subsequently reorganizes them selectively through frequency modulation, achieving multidimensional separation. This capability was demonstrated using ellipsoidal and spherical polystyrene particles, enabling shape-specific sorting at flow rates as high as 120 mL/h and allowing for shape-selective separation at high flow rates up to 120 mL/h, achieving a throughput 1000 times higher than similar microchannels with comparable efficiency [108]. Chu et al. described a two-step ODEP method for the purification of cell samples obtained by negative selection and immunomagnetic microbead-based isolation of circulating tumor cells (CTCs). The method uses the different responses of ODEP forces to cells bound to magnetic microbeads and cells not bound to magnetic microbeads to sequentially sort and isolate cancer cells from cells bound to microbeads using an ODEP-based virtual cell filter and tracker. This two-step ODEP cell processing technique increased the throughput and cell purity and resulted in significantly higher purity of cancer cells with purification efficiencies in the range of 81.6–86.1% compared to other ODEP-based CTC isolation techniques [109]. Zhang et al. proposed a microfluidic chip based on DEP for the rapid separation and high-purity purification of macrophages. They utilized DEP spectrum mapping under different electric field frequencies to identify specific frequencies that could effectively separate RAW264.7 macrophages from MCF-7 breast cancer cells and optimized the separation conditions in experiments by adjusting voltage and flow rate. The authors have successfully achieved macrophage separation with a purity of up to 99% using their chip and have been able to separate macrophages from plasma samples with a purity of 98% [110]. 

**Figure 11:**
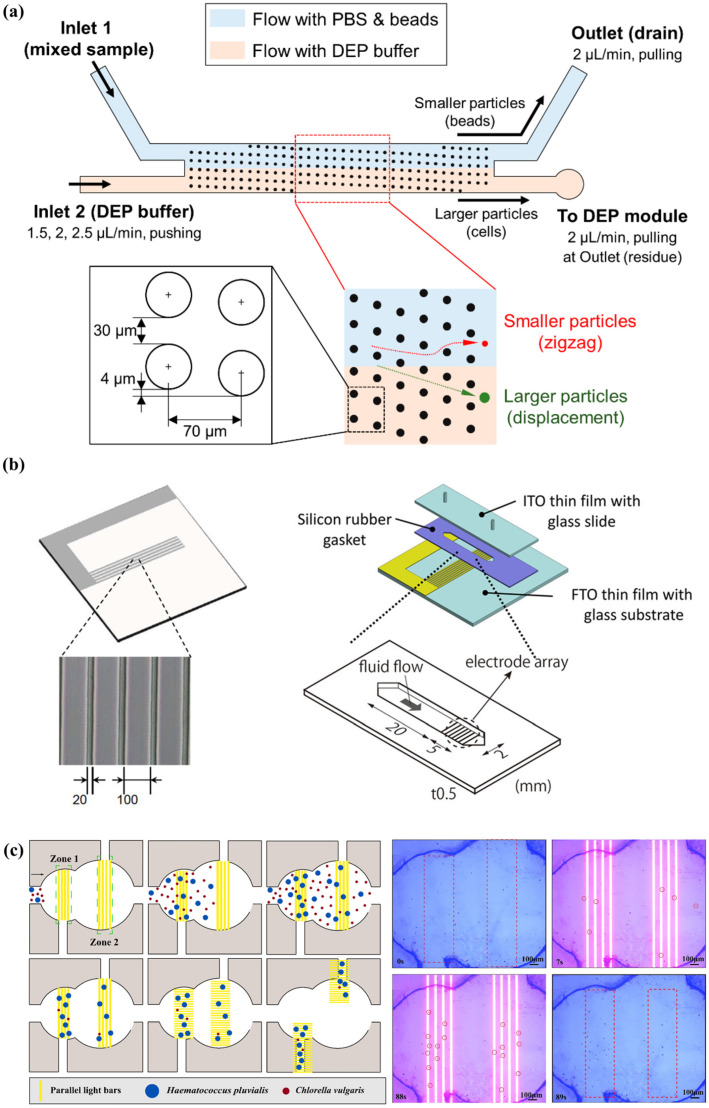
Schematic illustration of continuous purification of biological cells by utilizing (**a**) integrated hydrodynamic purification and dielectrophoretic trapping Reproduced from ref. [111]. Copyright 2020, MDPI, (**b**) the dielectrophoresis and flow-control system Reproduced from ref. [112]. Copyright 2022, Wiley, (**c**) optically-induced dielectrophoresis separation of microalgae by applying light patterns under 6 V and 100 kHz AC electric field. Reproduced from ref. [113]. Copyright 2024, AIP Publishing.

It can be seen from Figure 11 that Park et al. designed an integrated microfluidic system for the analysis of suspended cancer cells. The system combines a preprocessing module based on deterministic lateral displacement (DLD) and a DEP module utilizing an electroactive microwell array (EMA). Using an ordered array of pillars that manipulate particle paths based on their size enables effective size-based separation in the process. The DLD module is capable of separating prostate cancer cells (PC3) from a sample mixed with microbeads and exchanging the medium with the DEP buffer. The separated target cells from the DLD module are then introduced to the DEP module, where single-cell-level capture is realized by applying a positive DEP force through electrodes beneath the microwells. The authors successfully separated PC3 cells from a mixed sample using their system, with a cell separation efficiency of over 94% and a capture efficiency of over 93% for PC3 cells at a flow rate of 2.5 μL/min [111]. Kiryo et al. reported a method utilizing DEP and a flow management system to purify pluripotent stem cells (PSCs) based on their pluripotency status. They examined the dielectric properties of mouse embryonic stem cells (mESCs) with and without pluripotency, observing distinct frequency dependencies in their DEP responses. Leveraging these variations, the author established a cell sorting system capable of effectively segregating mESCs according to their pluripotency status, eliminating the need for fluorescent dyes or magnetic antibodies in the process. By employing DEP and a flow control system, they successfully separated mESCs based on their pluripotency, enriching the pluripotent cells in the collection port with a purification efficiency of approximately 90% [112]. Bian et al. reported a microfluidic chip based on optically induced DEP for achieving continuous purification of marine microalgae. The top layout of the microfluidic channels of the chip was designed with three different widths of 0.5 mm, 1.5 mm, and 2 mm for generating different flow conditions to achieve secondary tandem separation of *Haematococcus pluvialis*. They demonstrated a reduction of *Haematococcus pluvialis* from 37.5% to 1.2% after mixed liquid samples were collected at the chip exit under optimized ODEP operating conditions. High-quality purification of *Chlorella vulgaris* was achieved [113]. Chen et al. reported an iron/polyvinyltetrazole (pFeP/PVT) composite microsphere adsorbent incorporating DEP. They changed the dielectric properties of the pFeP/PVT composite microsphere adsorbent by adjusting the frequency of the electric field, utilized the generated dielectrophoretic force to attract the micelles of heavy metal ions to achieve high-efficiency adsorption at low frequencies, and generated repulsive force to promote the desorption of the micelles at high frequencies to achieve the recycling of adsorbents. The authors successfully achieved an increase in the removal rate of heavy metal ions from about 30% to about 90% using their device, and the adsorbent can be recycled up to 10 times [114].

### 3.4. Particle Focusing

**Figure 12:**
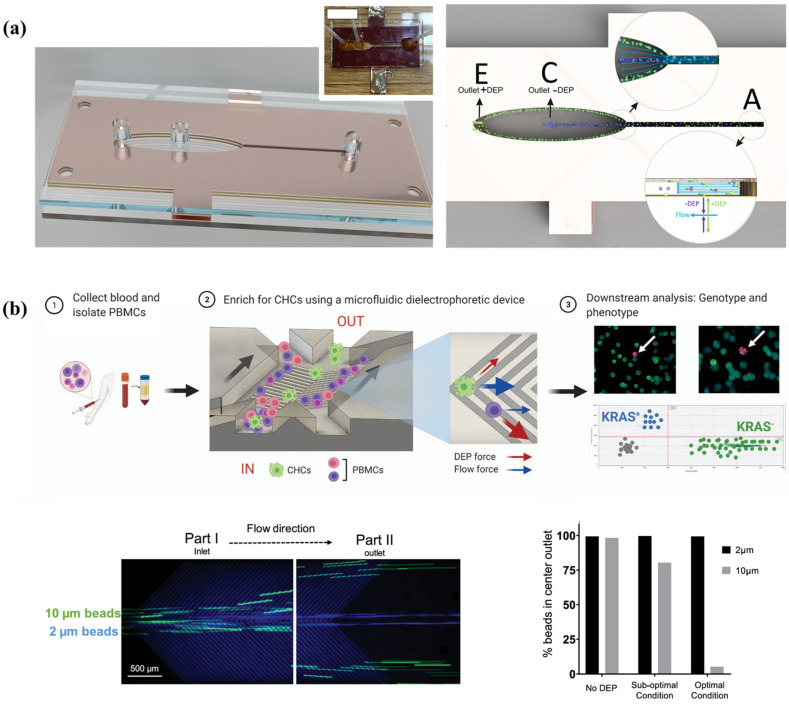
Schematic illustration of the focusing and enrichment of biological cells (**a**) in the 3D-integrated dielectrophoretic channels. Reproduced from ref. [115]. Copyright 2022, Wiley, (**b**) by a label-free dielectrophoretic microfluidic chip with deposited microelectrodes. Reproduced from ref. [116]. Copyright 2021, Springer Nature.

A three-dimensional integrated dielectrophoretic channel is proposed for continuous cell focus. They used a cut plotter to cut chips from a conductor-insulator laminate and were able to design and construct functional fluidic devices in a matter of hours at a fraction of the cost without the use of chemicals, masks, or clean room environments. Using their microchip, the authors successfully achieved enrichment of yeast cells to 80% purity at a rate of 10,000 cells per second with only about 12% cell loss [115]. Mira et al. designed a microfluidic device with interleaved electrode arrays for enrichment of rare circulating tumor cells, specifically circulating hybrid cells (CHCs), from blood. The device uses a specific V-shaped electrode structure that allows peripheral blood mononuclear cells (PBMCs) to be deflected by DEP forces in a densely populated cell environment, while CHCs remain in the central channel due to a weaker DEP response. Efficient depletion of PBMCs and enrichment of CHCs were achieved by optimizing voltage and frequency parameters. Their device achieved a 96.5% reduction in PBMCs and an 18.6-fold increase in tumor cell concentration. In pancreatic cancer patient PBMCs, the platform successfully enriched tumor cells harboring KRAS mutations using droplet digital PCR within 1 h, demonstrating enrichment in 75% of clinical specimens [116], as shown in Figure 12. 

Keck et al. investigated the use of DEP technology to enrich Trypanosoma brucei at specific locations. They designed a semicircular microelectrode that was fabricated on silicon wafers to generate a strong localized electric field gradient that helps to capture and enrich target cells at specific locations. It was also experimentally determined that under specific frequency (750 kHz) and voltage (5 Vp-p) conditions, the positive DEP response could be most effectively utilized to attract parasites to specific regions of the electrode. Ultimately, the authors achieved a 780% focus of parasites in a specific region within 50 s under optimal conditions [117]. Cao et al. developed a DEP-based technique for enhancing protein immunoassays with high sensitivity. They created Ag/SiO2 nanorod arrays using oblique angle deposition (OAD), generating ultrahigh-voltage electric field gradients. By applying bias voltages as low as 5 volts, they efficiently captured small proteins. Their method utilized oxide-coated silver nanorod arrays and prefabricated serrated electrodes, achieving a remarkable 1800-fold enhancement of bovine serum albumin in just 180 s. Using this setup, they demonstrated an ultrasensitive immunoassay for mouse immunoglobulin G, lowering the detection limit from 5.8 ng/mL to 275.3 femtograms/mL, significantly boosting sensitivity. Furthermore, they applied the technique to detect prostate-specific antigen cancer biomarkers in human serum, achieving a detection limit of 2.6 ng/mL. The enhanced sensitivity is attributed to the rapid biomarker enrichment and the metal-enhanced fluorescence effect enabled by the integration of nanostructures. Enhanced protein enrichment also accelerates binding kinetics, yielding saturated signals within one minute [118]. Nguyen et al. designed a microfluidic chip technology integrating DEP focus and impedance measurement for the detection of rare lung cancer circulating tumor cells (CTCs). They designed microfluidic devices containing circular microelectrodes that use positive DEP and hydrodynamic drag forces to guide the target cell (lung cancer cell A549) to the center of the working area and capture it on the desired sensing electrode. The presence of cells was identified by measuring impedance, and impedance spectroscopy was used to analyze different numbers of cells. Using their chip, the authors achieved over 90% enrichment of A549 cells on the sensing electrodes and were able to measure the impedance ringing of the cells captured on the electrodes in the frequency range of 1 kHz to 1 MHz [119]. 

### 3.5. Particle Assembly

The assembly behavior of segmented metal-dielectric particles under DEP effects is investigated. They fabricated segmented particles filled with gold (Au) and solvent using the templated electrodeposition technique. The synthesized particles were suspended in deionized water, placed on slides with microelectrodes, and then an AC electric field was applied to observe the DEP behavior of the particles. It was observed that certain segmented particle types rotated 90° as they neared the crossover frequency, reorienting their long axis perpendicular to the direction of the applied electric field. By exploiting the dielectrophoretic behavior of segmented particles in an AC electric field, they effectively achieved independent control over subpopulations of particles in binary mixtures composed of multicomponent particles with varying segmentation patterns [120]. Zhou et al. designed an atomic force microscopy probe-induced dielectrophoresis (AFM-DEP) chip for spatial manipulation and assembly of nanoparticles. Their chip combines the precise localization capability of AFM with the ability to manipulate particles in parallel with DEP. An AC voltage applied between the AFM probe and the ITO substrate generates a spatially non-uniform electric field. This facilitates precise control of nanoparticle manipulation and assembly directly on the chip. The probes act as movable DEP tweezers to manipulate and assemble nanoparticles by moving the probes. The authors have successfully utilized their AFM-DEP technique for the three-dimensional manipulation of nanoparticles. The effect of parameters such as gap distance, AC voltage, solution depth, solution concentration, and duration on nanoparticle assembly has also been investigated. The nanoparticles were assembled into basin shapes, linear structures, elliptical structures, and dot matrix structures by adjusting specific parameters [121]. 

Zheng et al. designed AC-coupled DEP to purposefully assemble single-walled carbon nanotubes (SWNTs) devices on a large scale. They added a suspension of SWNT droplets onto a curved, flexible substrate. An AC power supply was applied, and the SWNTs were subjected to dielectrophoretic forces in a non-uniform electric field, which attracted and deposited between the tips of the electrode pairs. After the first SWNT or SWNT bundle forms a reliable contact on the electrode pair, subsequent SWNT deposition becomes unfavorable as the first deposition changes the electric field distribution around the electrode pair, and self-limiting deposition is achieved. The authors confirmed the effect of self-limiting deposition by SEM and AFM imaging, as well as Raman spectroscopy analysis, which observed that the majority of the electrode pairs were bridged by single SWNTs or small bundles of SWNTs [122]. Frisenda et al. prepared TiS_3_ nanoribbons through liquid-phase exfoliation and utilized the dielectrophoretic (DEP) method to direct the assembly of these nanoribbons between two gold electrodes, thereby fabricating photodetectors operational within the visible light spectrum. Previous research has successfully utilized DEP for assembling carbon nanotubes or graphene [123,124,125,126]. They applied a 1 MHz sinusoidal signal to create a non-uniform electric field between the electrodes, using the DEP effect to attract the TiS_3_ nanoribbons to the area of maximum electric field intensity, achieving orderly arrangement and assembly between the electrodes. The resulting photodetectors exhibited a photocurrent responsivity of 3.8 mA/W at a 1 V bias under 405 nm blue light illumination, outperforming current devices that rely on two-dimensional materials exfoliated in liquid phase and assembled using drop-casting or ink-jet techniques [127]. 

**Figure 13:**
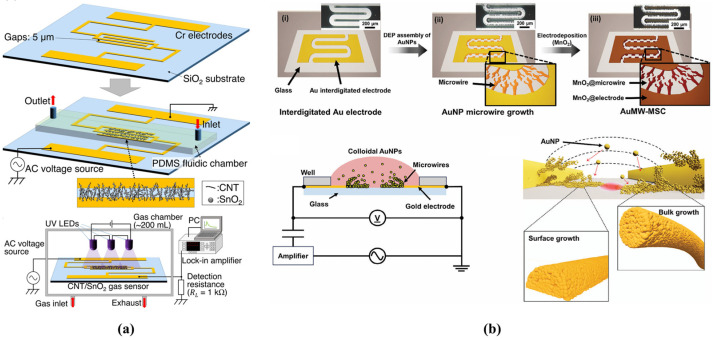
Schematic illustration of the dielectrophoretic assembly of (**a**) nanoparticles, Reproduced from ref. [128]. Copyright 2021, Elsevier. (**b**) nanoporous metal microwires. Reproduced from ref. [129]. Copyright 2024, Wiley.

The dielectrophoretic assembly of different nano-targets is shown in Figure 13. Inaba et al. utilized one-step dielectrophoretic (DEP) assembly technology to fabricate NO_2_ gas sensors that incorporate p-type carbon nanotubes (CNTs) and n-type tin dioxide (SnO_2_) nanoparticle heterojunctions. They varied the ratios of CNTs and SnO_2_ nanoparticles, suspended the mixture in deionized water, and used DEP force to assemble the particles into sensor channels at the gaps between electrodes. By adjusting the mixing ratios and DEP assembly times, they optimized the sensor performance. The sensors achieved a high response of approximately 1 ppm NO_2_ in a nitrogen environment and around 20 in synthetic air, with UV light used solely for initialization. Additionally, under continuous UV exposure, the sensors exhibited a maximum normalized response of about 19 to 1 ppm NO_2_ in synthetic air, detecting NO_2_ concentrations as low as 20 parts per billion [128]. Seo et al. reported a chip-scale micro-supercapacitor with high area energy density that utilizes nano-porous metallic microwires (AuMWs) formed by DEP-assembled gold nanoparticles (AuNPs) as electrodes. They applied an AC voltage to the interleaved finger electrodes, and the AuNPs aggregated in the electrode gaps due to the DEP force, forming this microwire structure. By electrodepositing MnO_2_ on the electrodes and AuMWs via cyclic voltammetry, combined with the porous rough surface of the AuMWs, a significant increase in the electrode surface area and enhancement of the capacitor’s charge storage capacity were achieved. The authors utilized their device to demonstrate a 72% and 78% improvement in specific capacitance and area capacitance for AuMW-MSCs compared to MSCs without integrated microwires [129]. 

## 4. Conclusions

In this paper, we highlight recent advances in the field of dielectrophoretic manipulation and separation of particles, especially the challenges posed by manipulating microparticles of various nanoscale sizes and materials. Dielectrophoretic manipulation relies on applying a force to polarizable materials within a nonuniform electric field. This technique encompasses two primary methodologies: direct current dielectrophoresis (DC-DEP) utilizing insulator-based approaches and alternating current dielectrophoresis (AC-DEP) employing micro-electrodes to create varying electric field distributions. Depending on the specific application and materials involved, static or dynamic fields with specific frequencies are utilized to ensure reliable operation in studies manipulating diverse particles. The electric field forces generated can be used for particle separation, capture, purification, focusing, assembly, etc. The non-contact, highly sensitive, and easy-to-use nature of DEP technology makes it more suitable for particle manipulation and separation. In addition, among the diverse techniques in other fields, we advocate combining microfluidic platforms with the high sensitivity and precision of DEP. We review recent advances in dielectric manipulation and separation of particles in three sections:

(1) In DC-DEP, a DC power supply and internally designed insulating structures are utilized to generate a non-uniform electric field within the chip, which enables the separation and manipulation of particles. DC-DEP is mainly categorized into electrode-based DC-DEP and insulator-based DC-DEP. Electrode-based DC-DEP relies on electrodes embedded in microfluidic channels to generate non-uniform DC electric fields. These electrodes can be simple two-dimensional planar structures, such as 2D planar electrodes formed on the bottom or other surfaces of the microchannels. They can also be more complex 3D structures, such as top-bottom patterned electrodes, sidewall patterned electrodes, and three-dimensional electrode structures formed by electroplating or chemical vapor deposition techniques. When a DC voltage is applied, these electrodes are capable of generating a non-uniform electric field directly in the microfluidic channel, enabling precise manipulation of the particles. Insulator-based DC-DEP, on the other hand, utilizes insulator structures to generate non-uniform electric fields without the need to embed electrodes directly in the microchannel. The technique can be embedded in microchannels with insulating barriers, such as circular or diamond-shaped pillars. Alternatively, the microchannels can be designed in serrated, spiral, or other curved shapes to utilize the geometry to create non-uniformity in the electric field. The use of insulators isolates the electrodes from the main channel and generates an electric field in the main channel through capacitive effects. This approach avoids direct contact between the electrode and the sample, reducing the risks of contamination and electrochemical reactions. Effective separation can be achieved at a lower voltage, thereby reducing the Joule heating effect and electrochemical reactions, which helps to preserve the integrity and activity of biological samples. Moreover, it also supports particle separation under continuous flow conditions, which not only improves separation efficiency but also increases throughput. This is particularly crucial for clinical and research environments that need to handle a large number of samples. Electrode-based DC-DEP provides greater manipulation precision and controllability of the electric field distribution, while insulator-based DC-DEP provides better biocompatibility and simplicity. In future developments, more functionalities, such as cell culture, chemical reactions, and biosensing, could be integrated to achieve comprehensive, one-stop biomedical analysis.

(2) AC-DEP utilizes a non-uniform electric field gradient generated by an AC electric field to exert a force on particles, enabling manipulation and separation of cells or particles. The core principle of the technique relies on the Clausius-Mossotti (CM) factor, which describes the relative capacitance and conductivity between the particle and the medium. When the real part of the CM factor is positive, the particles move towards regions with high electric field strength (pDEP); when the real part of the CM factor is negative, the particles move towards regions with low electric field strength (nDEP). The electrode structures of AC-DEP are crucial for realizing a non-uniform electric field, and they have a direct impact on the manipulation effect of the particles or cells. For example, the DEP effect can be enhanced by employing asymmetric electrode structures. Common AC-DEP electrode structures include planar electrodes, array electrodes, and fork-finger electrodes. Each structure is capable of generating a specific DEP effect in a non-uniform electric field according to its design features, which is suitable for different cell manipulations and separations. The strength and duration of the electric field have a significant effect on the cell manipulation effect and need to be optimized according to the experimental purpose. The choice of experimental conditions can improve the efficiency of cell manipulation and the accuracy of separation. By adjusting the frequency and intensity of the AC electric field, the manipulation and separation of different types of cells can be realized, such as the separation of live cells from dead cells. Frequency-modulated wave DEP can realize the periodic U-turn motion of cells in a short period of time and is used for the rapid measurement of crossover frequency. These electrode structures enable efficient electric field manipulation in smaller spaces, providing higher precision and sensitivity. The electrode designs are trending towards integrating multiple functions. Electrodes such as integrated DEP and AC insulator-based DEP can achieve more complex manipulation modes. This integration not only enhances operational flexibility but also broadens application ranges, such as in liquid flow, particle separation, and manipulation. High-density electrode array designs significantly improve the manipulation and parallel processing capabilities of electrodes. This design allows simultaneous processing of a large number of particles or cells in the same experiment, thus increasing both the efficiency of experiments and the speed of data acquisition. In recent years, DEP technology has made significant progress in the field of biomedical research, especially in applications such as manipulation and separation of microplastic particles, cell transfection, and microalgae harvesting, which have shown unique advantages.

(3) DEP is the movement of electrically polarized particles in a non-uniform electric field due to the interaction of their internally induced electric dipoles with the spatial gradient of the electric field. The combination of this technology with microfluidic chips provides an effective method for the sequential isolation of cells. The advantage of DEP lies in its non-invasive and labeling-free nature, which makes it possible to accurately differentiate and purify cells with different pluripotencies without causing damage to cells or biomolecules. In addition, DEP is characterized by low cost and rapid prototyping. Utilizing non-traditional microfabrication techniques, such as cutting plotters, researchers are able to design and build functional microfluidic devices in a very short period of time and at a very low cost. In the field of nanomanipulation, DEP is combined with atomic force microscopy (AFM). By applying an AC voltage between the AFM probe and a conductive substrate, a non-uniform electric field can be generated, enabling precise manipulation of nanoparticles. In recent years, DEP has made remarkable developments in a variety of applications. With its precise cell manipulation capabilities, DEP has successfully enabled the isolation of different types of cells, including cancer cells and stem cells. It has facilitated the capture and analysis of microscopic particles such as marine microplastics, microorganisms, and plankton. It has shown significant results in wastewater treatment, especially in the separation and recovery of suspended solids, oil droplets, and hazardous chemicals. We recommend the combination of microfluidic platforms with DEP for more applications.

With advancements in technology, DEP has significantly improved its precision for manipulating nanoscale samples. Recent research has demonstrated the ability to manipulate particles within the nanometer range, providing robust support for precise nanoscale operations. This high-resolution control capability is of great significance for the development of nanoscience and nanotechnology. Future research will increasingly focus on the application of functionalized nanomaterials in DEP. For instance, the use of composite materials such as magnetic nanoparticles and metal-coated nanoparticles can enhance the flexibility and effectiveness of DEP. These functionalized nanomaterials not only improve manipulation performance but also enable intelligent responses in specific environments, thereby expanding the range of applications for DEP technology. DEP technology performs well at the laboratory scale but faces challenges in large-scale production and industrial applications. Integrating other microfluidic functions such as mixing, separation, and detection into a single platform is a complex task that requires precise control of various microfluidic operations. To enhance scalability, flexible electronics, low-cost materials, and high-throughput microfluidic chips can be used. The development of novel materials and structures, digital microfluidics, and nanofluidics can improve integration. Establish standardized operating procedures and quality control measures to ensure the reproducibility and reliability of experiments and develop more accurate control systems and monitoring devices.

## Figures and Tables

**Table 1:** Comparison of different methods for the manipulation and separation of microparticles and biological cells.

Technique	Working Mechanism		Advantage	Limitations	Refs.
Acoustic	Acoustic radiation pressure transfers momentum from an acoustic wave to a particle		Large number of particles can be processed at the same time with high efficiency, wide operating range in the channel space, contactless operation, wide versatility, good biocompatibility, high precision, adjustable control, and flexible function	Relatively high equipment cost, need for frequency-specific acoustic sources, need for precise control of acoustic sources and microfluidic structures, design complexity, induced thermal energy increases temperature, relatively low throughput, problems associated with wavelength and diffraction	Zhang et al. [21], Gao et al. [22], Friend et al. [23]
Electrical	Electrphoresis (EP)	The electrophoretic force drives charged particles to move in the direction of the electric field	Suitable for rapid separation of charged particles, low voltage is sufficient for operation, simple equipment, easy to build and control, low cost	Limited to charged particles, the electric field conditions need to be optimized to avoid particle aggregation	Lomeli-Martin et al. [24], Zhang et al. [25]
	Dielectrophoresis (DEP)	Interactions involving the electrical polarization of particles and a non-uniform electrical field	Highly selective and sensitive to the electrical properties of particles, manipulating neutral particles, precise operation, label-free, real-time control, automated, microfluidic, and electronic compatible	Requires frequency-specific voltages, sophisticated electrode design, and complexelectric field control, higher cost, joule heat effect, low, and side effects affecting cell viability	Zhang et al. [26], Li et al. [27], Encinas [28], Kim et al. [29]
Magnetica	Homogeneous/inhomogeneous magnetic field		Magnetic particles can be manipulated for specific applications, high purity, highly specific cell separation based on magnetic labeling, or label-free cell manipulation based on negative magnetic electrophoresis	High cost of equipment, requires specific types of magnetic particles, additional cost of magnetic markers and magnetic fluids, relatively low throughput	Hejazian et al. [30], Giouroudi et al. [31], Pamme [32]
Optical	Manipulation of particles by radiation pressure exerted by a focused laser beam		Non-contact operation for precise manipulation of individual particles and high efficiency	Requires expensive laser systems and precision optical components with high alignment requirements, high equipment costs, may be damaging to particles, and requires complex optical system design	Gong et al. [33], Xie et al. [34]

**Table 2:** DEP manipulation and separation of microparticles and biological cells.

Method	Structure	Sample	Medium	Application	Flow Rate or Throughput	Efficiency or Purity	Ref.
DC-DEP	Sawtooth-shaped structure	HEK 293 cells, NSPCs	DEP buffer	Distinguishing and characterizing	-	>99.99%	Liu et al. [46]
	Non-uniform electric field generated at the tip of the microtubule	Small extracellular vesicles (30–150 nm)	Biofluid	Isolation	0.6 mL/h Throughput	>90%	Shi et al. [47]
	Sawtooth microchannel	Listeria monocytogenes	Phosphate buffer	Separation and identification	1.18 × 10^8^ bacteria/s/m^2^ Throughput	95%	Crowther et al. [48]
	Metal-Semiconductor-Metal	ZnO nanowires (NWs)	Zinc acetate, HMTA	Arrangement	1.28 A/s Forward Bias 20,000 A/s Reverse Bias	>90%	Sun et al. [49]
	Two electrically insulated columns with different clearances	Exosomes from MCF-7 cells (104.02 ± 6.99 nm)	Bidistilled water	Separation	0.3 mL/min Throughput	>90%	Ayala-Mar et al. [50]
	Asymmetric orifice	Chlorella (3 μm, 6 μm)	PBS	Separation	-	100%	Gao et al. [51]
	Asymmetric orifice	PS (3 μm, 4 μm, 6–7 μm)	PBS	Separation and counting	10–20 particle/min Throughput	>90%	Song et al. [52]
	Symmetric/Asymmetric ratchet	PS (3 μm, 5 μm, 10 μm)	PBS	Focusing	1.86 × 10^−8^ m^2^/(V·s)	Survival rate 98%	Lu et al. [53]
	Bifurcating microchannel	PS (5 μm, 15 μm)	PBS, 0.5% Tween 20	Separation	12 μL/h Throughput	nearly 100%	Li et al. [54]
	Asymmetric nano-orifice	PS (140 nm, 490 nm, 7 μm, 15 μm), magnetic nanoparticles (150 nm), magnetic-coated PS (470 nm, 5.2 μm), sliver-coated hollow glass beads (14 μm)	K_2_HPO_4_	Continuous separation	0.468 × 10^−4^ μL/s, 1.315 × 10^−3^ μL/s	-	Zhao et al. [55]
	Zigzag	PLT (1–5 μm), RBC (4–15 μm)	PBS	Separation	200 μm/s	>99.4%	Guan et al. [56]
	Nano-orifice	Oil droplet	KCl aqueous solution	Oil/water separation	175.2 µm^3^/s	-	Ren et al. [57]
	Dead-end branches	Droplet of fresh human blood	Blood plasma, RBCs	Blood plasma separation	0.857 μL/h Throughput	99%	Mohammadi et al. [58]
	Constricted channel region	Protein Crystals (100 nm–2.5 μm)	Pluronic F108 aqueous solution	Sorting	>70 μL/h	>90%	Abdallah et al. [59]
	Asymmetric orifice	PS (0.5 μm, 1 μm, 3 μm), Fluorescent (51 nm, 140 nm)	DI water, K_2_HPO_4_	Separation	4.758–6.717 μL/h	>90%	Zhao et al. [60]
AC-DEP	Nanogap electrodes	SSLBs, brain-derived myelin particles	DI water, PBS	Trapping and immobilization	10 μL/min	> 90%	Barik et al. [61]
	Inclined, comb-shaped electrodes	PS (8 μm, 10 μm, 12 μm) Bacillus cereus, S. aureus, *E. coli*, MCF7, Jurkat	CROSSORTERTM Buffer, PBS	Separation and enrichment	1–2 mL/h	92.3%	Oshiro et al. [62]
	Asymmetrical aluminum electrodes	Tetraselmis sp.	Artificial seawater medium	Harvesting of microalgae biomass	2.5 mL/min	90.9%	Hawari et al. [63]
	Microelectrode Needles	T cell (10–15 μm), B cell (7.5–10 μm), MLV	DI water, Sucrose solution	Directed Movement, Periodic U-Turns, Trapping, and Release	5 μL/min	>90%	Frusawa et al. [64]
	Triangular ratchets	PS (3 μm, 5 μm, 10 μm), yeast cells (7 μm)	PBS	Focusing and separation	144 μm/s	90%	Malekanfard et al. [65]
	Interdigitated gold electrodes	PS (3 μm, 5 μm, 10 μm)	PBS, sucrose, etc.	Focusing and separation	40 μL/h	PS: 98.7%, MCF7: 82.2%	Modarres et al. [66]
	Dual electrodes	PS (10 μm), HEK-293	Sucrose solution	Cell capture and electroporation transfection	20–140 nL/min	80%	Punjiya et al. [67]
	Transparent parallel-line electrode array	MESCs (5–8 µm), MEFs (10–20 µm)	LCB, HEPES, CaCl_2_, sucrose solution	Separation	24 μL/min	90%	Takahashi et al. [68]
	Circular channel with electrodes on the sidewalls	PS (2 μm, 3 μm, 3.5 μm), RBCs, WBCs, MDA-MB-231	PBS	Separation	200 μm/s	-	Derakhshan et al. [69]
	Nanogap Electrodes	AuNW	Gold Nanowire Suspension	Single Nanowire Assembly	-	70%	Han et al. [70]
	Y-Y shaped microchannel, alternating triangular electrodes	NSCLC, RBC (5 μm), CTCs	Blood sample, Buffer solution	Separation of CTCs	200 μm/s	99%	Zhang et al. [71]
	Y-Y microfluidic	RBC, CTCs	DEP buffe	Isolation of CTCs from PB	200 μm/s	100%	Lv et al. [72]
	Stainless-steel wire mesh electrodes	Anabaena	Artificially prepared eutrophic water	Capture and removal of Anabaena algae	0.168–0.838 L/h	89.79%	Liu et al. [73]
	AC Insulator-based DEP	DNA	PBS	DNA Size Separation	1.3 μL/h	92%	Jones et al. [74]
	3D self-assembled ionic liquid electrodes	PS, PC-3, live cells, dead cells, ADSCs, and MDA-MB-231	DEP buffe	Separation	15 μL/h	PS, PC-3: 94.7%, live/dead cells: 89.8%, ADSCs: 81.8%, MDA-MB-231: 82.5%	Sun et al. [75]
	Four-sector electrode array	PS (50 μm)	DI water	Positioning, and Aggregation, Separation	200 μm/s	>90%	Zemánek et al. [76]
	BPE	CTCs	Buffer	Separation	0.1 mL/h	>80%	Li et al. [77]
	Microwells	CT26, BMDC	Buffer	Cell pairing and fusion	2.5 μL/min	86%	Pendharkar et al. [78]
	Porous Ni@PVDF conductive membrane	SiO_2_, Al_2_O_3_, BaTiO_3_	DI water, NaCl	Membrane antifouling	-	90.1%	Liu et al. [79]
	Asymmetric Orifice	Yeast cells	DI water, K_2_HPO_4_	Continuous cell characterization and separation	13.5 μL/h	-	Zhao et al. [80]
	3D electrodes	Chlorella (3–5 μm), Closterium	PBS	Separation	300 μm/s	>90%	Wang et al. [81]
	Right-angle bipolar electrodes	Euglena, H. pluvialis, C. reinhardtii, Dunaliella salina, and Platymonas	DEP buffe	High-efficiency selection of non-spherical flagellate algae	54 μL/h 72 μL/h 48 μL/h	92.06% 92.78% 99.06%	Chen et al. [82]
	Integrated DEP and inertial forces	Cladocopim (10 μm), Effrenium (15 μm)	PBS	Separation and enrichment	200 μm/min, 300 μm/min	90%	Zhou et al. [83]
	Microelectrodes	Yeast cells	TES, CaCl_2_, sucrose	Capture and separation	1 μL/min	>94%	Julius et al. [84]

## Data Availability

Not applicable.
